# A systems approach evaluating the impact of SARS-CoV-2 variant of concern mutations on CD8+ T cell responses

**DOI:** 10.1093/immadv/ltad005

**Published:** 2023-03-15

**Authors:** Paul R Buckley, Chloe H Lee, Agne Antanaviciute, Alison Simmons, Hashem Koohy

**Affiliations:** Medical Research Council (MRC) Human Immunology Unit, MRC Weatherall Institute of Molecular Medicine (WIMM), John Radcliffe Hospital, University of Oxford, Oxford, UK; MRC WIMM Centre for Computational Biology, Medical Research Council (MRC) Weatherall Institute of Molecular Medicine, John Radcliffe Hospital, University of Oxford, Oxford, UK; Medical Research Council (MRC) Human Immunology Unit, MRC Weatherall Institute of Molecular Medicine (WIMM), John Radcliffe Hospital, University of Oxford, Oxford, UK; MRC WIMM Centre for Computational Biology, Medical Research Council (MRC) Weatherall Institute of Molecular Medicine, John Radcliffe Hospital, University of Oxford, Oxford, UK; Medical Research Council (MRC) Human Immunology Unit, MRC Weatherall Institute of Molecular Medicine (WIMM), John Radcliffe Hospital, University of Oxford, Oxford, UK; MRC WIMM Centre for Computational Biology, Medical Research Council (MRC) Weatherall Institute of Molecular Medicine, John Radcliffe Hospital, University of Oxford, Oxford, UK; Medical Research Council (MRC) Human Immunology Unit, MRC Weatherall Institute of Molecular Medicine (WIMM), John Radcliffe Hospital, University of Oxford, Oxford, UK; Medical Research Council (MRC) Human Immunology Unit, MRC Weatherall Institute of Molecular Medicine (WIMM), John Radcliffe Hospital, University of Oxford, Oxford, UK; MRC WIMM Centre for Computational Biology, Medical Research Council (MRC) Weatherall Institute of Molecular Medicine, John Radcliffe Hospital, University of Oxford, Oxford, UK; Alan Turing Fellow in Health and Medicine

**Keywords:** SARS-CoV-2, CD8 T cell response, immunogenicity, T cell recognition potential, systems immunology, machine-learning

## Abstract

T cell recognition of SARS-CoV-2 antigens after vaccination and/or natural infection has played a central role in resolving SARS-CoV-2 infections and generating adaptive immune memory. However, the clinical impact of SARS-CoV-2-specific T cell responses is variable and the mechanisms underlying T cell interaction with target antigens are not fully understood. This is especially true given the virus’ rapid evolution, which leads to new variants with immune escape capacity. In this study, we used the Omicron variant as a model organism and took a systems approach to evaluate the impact of mutations on CD8+ T cell immunogenicity. We computed an immunogenicity potential score for each SARS-CoV-2 peptide antigen from the ancestral strain and Omicron, capturing both antigen presentation and T cell recognition probabilities. By comparing ancestral vs. Omicron immunogenicity scores, we reveal a divergent and heterogeneous landscape of impact for CD8+ T cell recognition of mutated targets in Omicron variants. While T cell recognition of Omicron peptides is broadly preserved, we observed mutated peptides with deteriorated immunogenicity that may assist breakthrough infection in some individuals. We then combined our scoring scheme with an *in silico* mutagenesis, to characterise the position- and residue-specific theoretical mutational impact on immunogenicity. While we predict many escape trajectories from the theoretical landscape of substitutions, our study suggests that Omicron mutations in T cell epitopes did not develop under cell-mediated pressure. Our study provides a generalisable platform for fostering a deeper understanding of existing and novel variant impact on antigen-specific vaccine- and/or infection-induced T cell immunity.

## Graphical Abstract

**Graphical Abstract:**
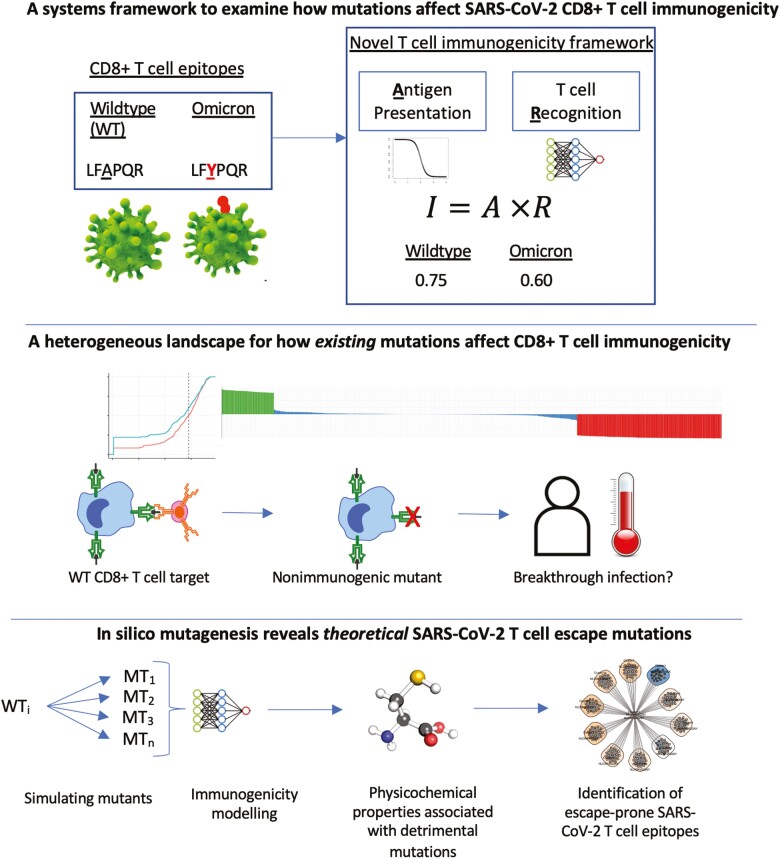

## Introduction

Cellular and humoral responses are pillars of adaptive immunity following vaccination or natural SARS-CoV-2 infection. A clear understanding of how emergent variants of concern (VOCs) affect adaptive immunity is essential for effective control of the pandemic, especially for a virus with such dynamic evolution, which threatens a future VOC with additional escape capacity.

Upon its emergence, the SARS-CoV-2 VOC Omicron BA.1 caused global concern due to alarming mutations in its surface glycoprotein. It, however, became clear that Omicron and derivative subvariants BA.2, 4, 5 among others are broadly associated with milder disease outcomes than previous strains [1]. Nevertheless, their higher transmissibility rates [2] contributed to widespread infection, social disruption, and healthcare turbulence.

While numerous studies have illustrated extensive humoral immune escape by Omicron and its subvariants [3–6], accumulating reports indicate that broadly, mutations in Omicron and other current VOCs do not drastically hinder T cell responses [7, 8]; likely due to the diversity of human leukocyte antigens (HLAs) and the breadth of epitopes targeted by T cells. To this end, a series of studies focused on memory T cells have shown that impact of variant mutations on T cell response is limited and the majority of CD4+ and CD8+ T cell responses are preserved in most naturally infected and/or vaccinated individuals [9–15]. For Omicron specifically, Tarke *et al.* reported preservation of at least 84% and 85% for CD4+ and CD8+ cell response respectively following a range of different vaccines, whereas a highly significant decrease for memory B cells was observed [16]. These studies have offered great insights that have informed policymaking on mitigation of the disease, e.g. booster rollouts.

Nevertheless, multiple studies have demonstrated substantial impairment in some cases [17, 18]. Indeed, Naranbhai *et al.* found that in ~20% of individuals (*n* = 10) examined, >50% of their T cell responses were impaired after Omicron [17]. More recently, Reynolds *et al.* found that individuals infected in the first wave and subsequently with Omicron had particularly poor T-cell responses [18] and that Omicron BA1 was a poor booster of immunity to subvariant Omicron infections. Furthermore, Suryawanshi *et al.* found that infection and vaccination ‘hybrid’ immunity does not appear to protect against different variants [19]. Indeed, such data are beginning to demonstrate complex heterogeneity underpinning whether breakthrough infections occur; and that the extent to which mutations from VOCs can impact T cell immunity in individuals is varied.

Adding to this heterogeneous landscape is that although many individuals now experience COVID-19 with mild symptoms, there are increasing reports of complications following SARS-CoV-2 infection. For example, considerable numbers of individuals suffer from post-acute sequelae of COVID-19 [20–22] (long COVID) and there are increasing reports of multisystem inflammatory disorders where SARS-CoV-2 has been implicated as a causative agent [20, 23–25]. Indeed, while T cells are thought to provide a barrier against Omicron infection where antibody responses are evaded, there is increasing evidence that T cells may, on the other hand, contribute to post-COVID complications in some individuals, e.g. through provoking inflammatory disorders [20, 23, 26, 27], elevated T cell exhaustion in long COVID [21] and CD8+ T cell infection and lymphopenia following SARS-CoV-2 exposure [28].

Collectively, these studies raise diverse questions regarding heterogeneity underpinning how T cells may protect from or exacerbate COVID-19 and related diseases. One such question is how mutations in VOCs such as Omicron have impacted the landscape of SARS-CoV-2-specific T cell responses. Additionally, the high prevalence of humoral and cellular immunity from vaccination and/or natural infection may impose selection pressures promoting new variants, threatening a future strain characterised by T-cell escape. Taken together, these studies demonstrate it is vital to understand how mutations from existing VOCs such as Omicron – and theoretical mutations – affect T cell responses to SARS-CoV-2.

Multiple studies have begun to address this gap [8, 29–31]. For example, Nersisyen *et al.* [29] recently compared all theoretical HLA ligands from the Wuhan Hu-1, Delta and Omicron proteomes. The authors concluded that most HLA alleles and theoretical major histocompatibility complex (MHC) haplotypes are not significantly affected by mutations in Delta/Omicron; therefore, effective T cell immunity is likely to be maintained. Notwithstanding, the authors reported a reduction in ‘tightly binding’ spike-derived HLA-B*07:02 ligands for both Delta and Omicron and an elimination of ligands for HLA-DRB1-03:01. Indeed, studies to date have generally focused on comparing antigen presentation of HLA ligands across VOCs. However, while antigen presentation is *required* for T cell immunogenicity, it is not *sufficient*. Therefore, to foster a detailed understanding of how mutations can affect SARS-CoV-2-specific T cell responses, it is vital to examine the subset of MHC-bound ligands that are known to invoke T cell activation.

Dolton *et al.* recently argued that the range of potential mutational impact on SARS-CoV-2-specific T-cell recognition is poorly understood and urgently requires further study [32]. Indeed, despite the great insights from the above research, a systematic study to examine the impact of mutations at all known SARS-CoV-2-specific CD8+ T cell targets, on T cell immunogenicity has not yet been reported. Therefore, there is an unmet need for research to evaluate and infer the impact of current and theoretical future mutations on T (and B) cell immunity.

**Figure 1.**
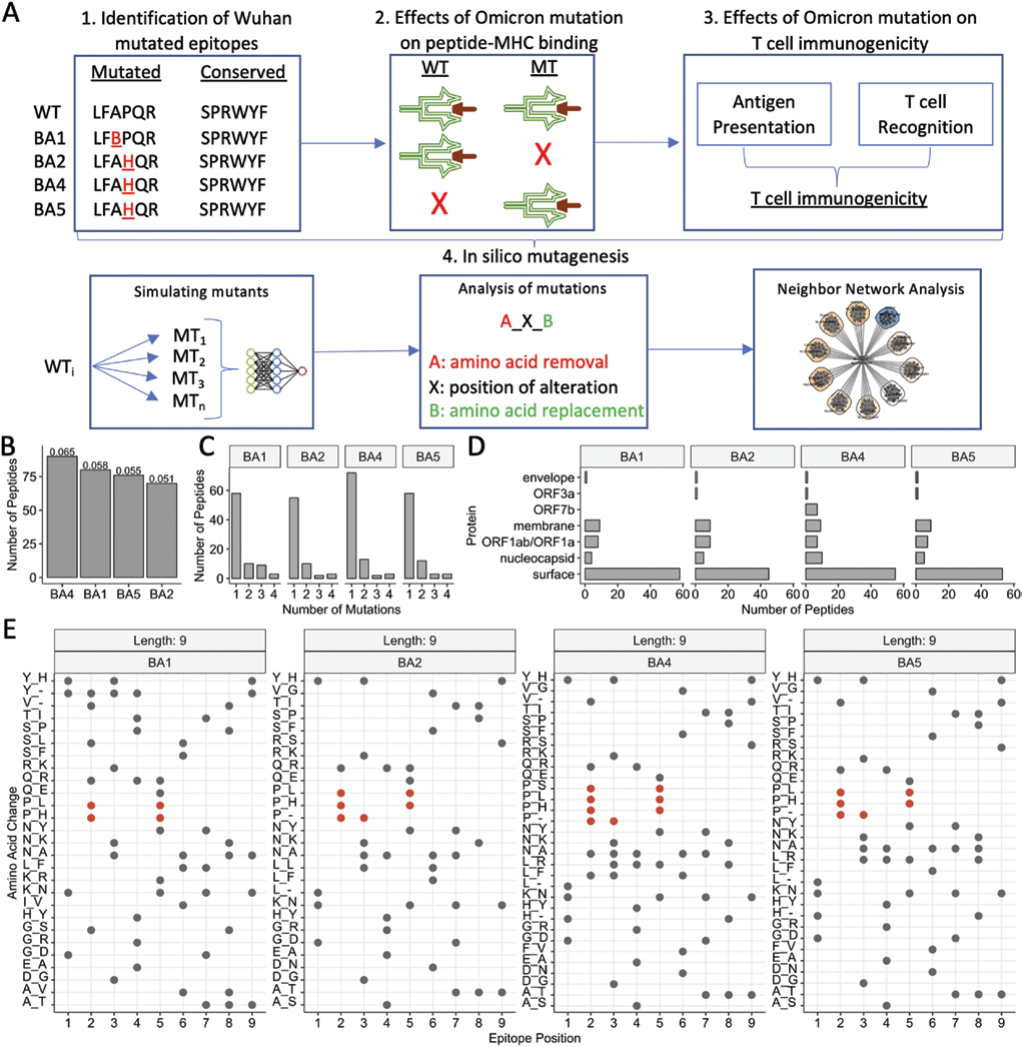
Study overview and identification of SARS-CoV-2-specific CD8+ T cell targets with mutation(s) in Omicron and its subvariants. (A) Overview of the study: (1) First, we identified the set of ‘Wuhan-mutated’ epitopes: CD8+ T cell targets from Wuhan Hu-1 with mutations in Omicron (BA.1) or its subvariants (BA.2, BA.4, and BA.5). (2) Using netMHCpan 4.1, we analysed the effects of mutations from aforementioned variants of concern on antigen presentation, through comparing predicted binding metrics (affinity *nM*, normalised binding affinity rank score) between Wuhan Hu-1 variant CD8+ T cell epitopes and counterpart mutant epitopes. To appropriately analyse potential bi-directional changes in antigen presentation between wildtype and mutant peptides, we incorporated wildtype-mutant (WT-MT) paired samples where (top) both WT and MT bind MHC *k*, where (middle) WT but not MT binds MHC *k*, and where (bottom) WT was not a ligand for MHC *k* in Wuhan-Hu-1 but the MT now binds MHC. (3) Next, we analysed the effects of the existing variant of concern mutations on T cell immunogenicity. T cell immunogenicity incorporates two key components: antigen presentation and T cell recognition. We, therefore, combined two scores for this analysis: (i) antigen presentation – generated by netMHCpan 4.1 – and (ii) T cell recognition, generated by an in-house deep learning model ‘TRAP’. (4) We next performed a comprehensive *in silico* mutagenesis to examine the effects of theoretical mutations on T cell immunogenicity. To do this, we first simulated each mutant (MT_1…_MT_*n*_) that can be generated via single-point substitutions for each WT Wuhan-mutated peptide of interest (WT_i_). For each wild-type and mutant peptide, we generated an immunogenicity score. Next, we analysed the effects of mutation on immunogenicity, through analysing WT-MT paired changes in immunogenicity score. In this article, we follow nomenclature where a ‘full’ mutation comprises three components: ‘A_*x*_B’. Here, residue ‘A’ is removed from epitope sequence position *x* and is replaced with residue ‘B’. Finally, we adopted the ‘neighbour-network’ framework of Ogishi et al. to visualise the effects of different residue substitutions, to help identify the most escape-prone mutations and understand how different mutations in different positions affect each epitope. (B) The number of CD8+ T cell targets that exhibit at least one mutation from BA.1 Omicron and/or derivative subvariants BA.2, BA.4, and BA.5. Numeric labels show the frequency of mutated CD8+ T cell epitopes, given a particular variant compared with the total number of assessed CD8+ T cell targets (*n* = 1380). (C) The distribution of the number of mutations found in CD8+ T cell targets across assessed variants of concern (with respect to Wuhan Hu-1). (D) The distribution of originating proteins for SARS-CoV-2 Wuhan Hu-1 CD8+ T cell targets with a mutation in each variant. (E) The landscape of amino acid substitutions across different variants of concern within CD8+ T cell targets of length nine residues. ‘X_Y’ on the y-axis indicates the removal of amino acid X, which is replaced by amino acid Y. The x-axis shows the position in the epitope where the mutation is observed. Red points highlight amino acid alterations which remove a ‘proline’.

In this study, using SARS-CoV-2 CD8+ T cell epitopes as a model system, we present a systematic framework to evaluate the impact of mutations at T cell targets on their immunogenicity (Fig. 1A). To model peptide immunogenicity, we consider (i) antigen presentation predictions using netMHCpan, and (ii) T cell recognition by leveraging a deep neural network workflow we have recently developed [33] for accurate predictions in this setting (Fig. 1A). By inferring the effects of Omicron mutations on CD8+ T cell epitopes, we found a heterogeneous landscape of impact, encompassing the most likely targets for T cell escape, as well as pMHC with potentiated immunogenicity following mutation. We then present an *in silico* mutagenesis and dissect the impact of theoretical substitutions on SARS-CoV-2-specific CD8+ T cell responses. Our approach allows a systematic evaluation of the impact of mutation on T cell responses, not only for those conferred by existing variants such as Omicron but also theoretical mutations. We believe that such an approach will evolve into pipelines to predict the impact of emerging variants on T cell immunity, e.g. by estimating the probability of T cell immune escape. These concepts could additionally be applied to other diseases that are affected by pathogenic mutations.

## Results

### The subset of Wuhan Hu-1 CD8+ T cell epitopes with mutation in Omicron

To investigate the effect of existing mutations on SARS-CoV-2-specific CD8+ T cell epitopes, we curated a pool of 1380 unique SARS-CoV-2 peptides from epitope databases that have been functionally evaluated for CD8+ T cell response (see ‘Methods’ section). Of these, 9-mers were most common (~54%), followed by 10-mers (25.6%) (Supplementary Fig. S1A).

To identify immunogenic Wuhan Hu-1 epitopes which are mutated in the parent Omicron variant BA.1 and subvariants BA.2, BA.4 and BA.5, we mapped our pool of 1380 peptides to each respective proteome (see ‘Methods’ section). We observed that BA.4 produced the greatest number of mutations in unique Wuhan Hu-1 CD8+ T cell targets (90/1380, ~6.5%), followed by BA.1 (80/1380, ~5.8%), BA.5 (76/1380, ~5.5%), and BA.2 (70/1380, ~5%) (Fig. 1B). For each variant, most of these alterations were single-point mutations, although epitopes with two or three mutations were also relatively abundant (Fig. 1C and Table 1). Unsurprisingly, for each variant, most CD8+ T cell epitopes that exhibited mutations were derived from spike glycoprotein (Fig. 1D).

**Table 1.** The number of epitopes (#) with *x* number of mutations in CD8+ T cell regions of variants BA.1, BA.2, BA.4, and BA.5, with respect to Wuhan Hu-1

# Mutations in epitope	Variant	#
1	BA.1	58
1	BA.2	55
1	BA.4	72
1	BA.5	58
2	BA.1	10
2	BA.2	10
2	BA.4	13
2	BA.5	12
3	BA.1	9
3	BA.2	2
3	BA.4	2
3	BA.5	3
4	BA.1	3
4	BA.2	3
4	BA.4	3
4	BA.5	3

Leveraging our dataset of Wuhan Hu-1 immunogenic epitopes with a mutation in Omicron strains (the Wuhan Hu-1 wildtype will hereby be referred to as ‘Wuhan-mutated’) and their respective variant counterparts, we explored alterations at different sequence positions across 9-mer (Fig. 1E) and 10-mer (Supplementary Fig. S1B) peptides. We observed P→L/H mutations at P2 of 9-mers among all variants. BA.4 poses an additional P→S mutation in this position. For 10-mers, we also observed proline substitutions, although they were less common (Supplementary Fig. S1B).

Proline substitutions are of particular interest, as a P→L mutation (P4, spike:YLQPRTFLL-HLA-A02: in non-Omicron variants) has recently been shown to escape SARS-CoV-2-specific CD8+ T cell responses [32]. Furthermore, a report by Hamelin *et al.* found that proline substitutions arise from mutational bias in SARS-CoV-2 evolution, which compromised binding of HLA-B07 ligands [34]. Interestingly, enzymes that are thought to drive the underlying nucleotide mutation (C→U), have been implicated in altering known HIV-1 epitopes. Taken together, mutations in globally disseminated VOCs are consistent with alterations that are known to compromise T cell epitopes.

### Effects of Omicron mutations on peptide-MHC-I binding

While antigen presentation by MHC is not sufficient for an effective T cell response, it is a prerequisite for T cell recognition. We, therefore, used the known CD8+ T cell targets in Wuhan Hu-1 to systematically study the impact of existing mutations on their presentation status. In this section, focusing on immunogenic Wuhan Hu-1 CD8+ T cell epitopes with alterations in Omicron variants, we report the effects of mutations on their capacity to bind MHC.

We first predicted HLA binding metrics (see ‘Methods’ section) of all immunogenic Wuhan Hu-1 SARS-CoV-2 epitopes to 64 HLAs which (i) are commonly observed in epitope datasets and (ii) have previously been employed by the ‘TCoV’ pipeline to compare overall MHC binding of SARS-CoV-2 variants [29]. As we have shown, such a strategy can identify MHC ligands with 98% accuracy [2]. Here, HLA-A*29:02 is predicted to present the most Wuhan Hu-1 CD8+ T cell targets (309/1380), while HLA-B*27:05 is predicted to present the least (35/1380) (full predictions are available in Supplementary data 1).

**Figure 2.**
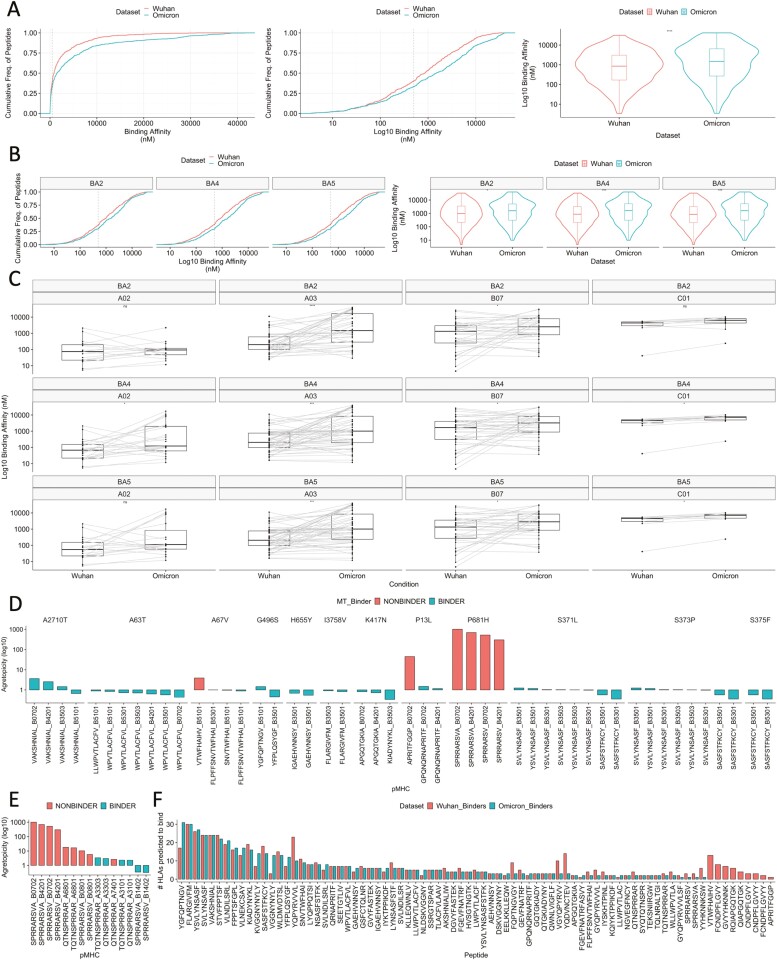
The impact of BA.1 Omicron and subvariant mutations on HLA binding of mutated SARS-CoV-2 CD8+ T cell targets. HLA binding predictions were made using netMHCpan 4.1. We analysed any paired wildtype (WT) or mutant (MT) samples where *either* the WT or the MT were predicted to bind MHC, to incorporate retained binding status and bi-directional transitions between WT and MT. Analyses were performed for the set of Wuhan Hu-1 CD8+ T cell targets with a mutation and their mutated counterparts. (A) empirical cumulative distribution (ECDF) plot showing (i) the cumulative frequency of peptides given the range of binding affinities (nM) for Wuhan-mutated vs BA.1 Omicron pMHC, (ii) the cumulative frequency of peptides, given the range of log10 transformed binding affinities (nM) for Wuhan-mutated vs BA.1 Omicron pMHC. (iii) Violin plots comparing the distribution of binding affinities (nM) between Wuhan-mutated pMHC and BA.1 Omicron mutants. Significance was assessed using a Wilcoxon rank test. Dashed line shows 500 nM. (B) (left) log10 transformed ECDF plots and (right) violin plots comparing the binding affinity (nM) of Wuhan-mutated vs. mutant pMHC, where the mutated epitope is derived from variants BA.2, BA.4, and BA.5. (C) Paired boxplots for different HLA supertypes (HLA-A02, HLA-A03, HLA-B07, and HLA-C01), comparing the binding affinities (nM, log 10) between Wuhan-mutated and mutant pMHC where the mutated epitope is derived from variants BA.2, BA.4, and BA.5. Significance was assessed using paired Wilcoxon rank tests. (D) Barplots showing log 10 scaled agretopicities (MT nM/WT nM), for BA.1 Omicron mutations affecting selected HLA-B07 supertype pMHC. Agretopicity > 0 indicates a detriment in HLA binding for the mutant compared with the wild type. Plots are colour labelled by whether the mutant pMHC is predicted to bind MHC or not (red). Red bars, therefore, indicate those mutant CD8+ T cell pMHC predicted to no longer bind MHC following variant mutation. (E) Barplots showing log10 scaled agretopicities for BA.1 mutations within CD8+ T cell targets that contain the spike glycoprotein motif ‘PRRA’. ‘PRRA’ is a motif unique to SARS-CoV-2, which is hypothesised to form a portion of a putative core of a theoretical SARS-CoV-2 superantigen. Plots are colour labelled by whether the mutant pMHC is predicted to bind MHC or not (red). (F) A barplot showing the number of HLAs predicted to bind each mutated wildtype epitope (labelled: Wuhan_Binders) vs. the Omicron BA.1 counterpart (labelled: Omicron_Binders).

Expanding this approach, we next compared the antigen presentation status of *Wuhan-mutated* and their BA.1, BA.2, BA.4, or BA.5 Omicron counterpart peptides against these 64 HLA-I alleles (see ‘Methods’ section). By comparing 463 spike-derived, paired wild-type (WT) vs. mutant (MT) predicted pMHC (57 peptides), we observed that BA.1 Omicron mutants were predicted as weaker binders (higher nM) to MHC-I alleles than their Wuhan Hu-1 counterparts (Fig. 2A). Different HLAs bind their ligands in different nM ranges, thus by analysing *netMHCpan* rank scores we confirmed these results are not due to HLA biases in the dataset (Supplementary Fig. S2A). We found a similar albeit weaker trend after comparing CD8+ T cell targets across *all* SARS-CoV-2 proteins (Supplementary Fig. S2B). We also observed that BA.2, BA.4, and BA.5 mutants also exhibited weaker predicted binding to MHC-I compared with Wuhan Hu-1 (Fig. 2B and Supplementary Fig. S2C and D).

By grouping paired observations by HLA supertype, we observed that binding affinity for BA.1 spike-derived B07-pMHC is perhaps weaker compared to Wuhan Hu-1 pMHC, although this effect was not significant (Supplementary Fig. S2E). For BA.2 and BA.5, we found that ligands bound to HLA-A03 and HLA-B07 were significantly impaired (Fig. 2C), with the addition of HLA-A02 for BA.4. By visualising supertype-specific footprints, we observed subtle differences in how peptides establish binding to these HLA supertypes (Supplementary Fig. S2F). As ~25–35% of the global population possess an A02, A03, or B07 supertype allele [34, 35], it is plausible that binding detriments for particular pMHC may lead to impairments in T cell reactivity for individuals carrying certain HLA, with differences across subvariants. Our data are consistent with Hamelin’s [34] arguments that mutational biases may shape SARS-CoV-2 T cell reactivity, and we show that mutations from globally spread VOCs in B07-binding CD8+ T cell target regions are predicted to significantly weaken pMHC binding affinity.

Focusing on B07-pMHC with mutations in the original BA.1 strain, we observed that the overlapping spike-derived ligands ‘SPRRARSVA’ and ‘SPRRARSV’ are most detrimentally affected (Fig. 2D). These CD8+ T cell targets are impacted by a P→H mutation in the P2 anchor position, consistent with Hamelin’s work [34]. The Wuhan Hu-1 sequences of these epitopes contain ‘PRRA’, which is a motif forming a core of a hypothesised and controversial SARS-CoV-2 ‘superantigen’ [26, 27]. This so-called ‘SARS-CoV-2 superantigen’ has been hypothesised to play a role in post-COVID-19 multisystem inflammatory syndrome [20, 23], although a recent study found no evidence for its ‘intrinsic superantigen-like inflammatory activity’ [36, 37]. In fact, 9/15 (60%) pMHC-I containing this hypothesised superantigen core are predicted to become ‘nonbinders’ as a result of BA.1 mutation (Fig. 2E and Table 2). Given the global spread of Omicron and its subvariants, these data suggest that CD8+ T cell responses induced by this hypothesised superantigen would currently be limited to few HLA (HLA-A*33:03, -A31:03, and -B14:02) which remain as presenters following Omicron mutation.

**Table 2.** CD8+ T cell targets containing the motif ‘PRRA’ with a mutation in Omicron BA.1 (with respect to Wuhan Hu-1). ‘PRRAR’ motif is hypothesised to be a key insert of a core of a theoretical SARS-CoV-2 ‘superantigen’. ‘Peptide’ lists the wildtype Wuhan Hu-1 CD8+ T cell target, ‘variant peptide’ lists the corresponding identified mutant in BA1, ‘HLA allele’ lists for each peptide variant, HLAs where either the wildtype peptide or the mutant (variant peptide) is predicted to bind, ‘WT_BA’ and ‘MT_BA’ shows the predicted binding affinity of the wildtype and mutant peptides, respectively. ‘Binder’ and ‘MT_Binder’ show whether the wildtype or mutant is predicted to bind the listed HLA (determined by binding affinity rank threshold output by netMHCpan 4.1). ‘Agretopicity’ shows the differential agretopicity index, the ratio (MT BA/WT BA) of mutant to wildtype binding affinities. An agretopicity > 1 indicates an impairment in binding affinity for the mutant compared with the wildtype while agretopicity <1 indicates the wildtype binds with lower affinity

Peptide	Variant peptide	HLA allele	WT_BA	MT_BA	Binder	MT_Binder	Agretopicity (MT/WT)
SPRRARSVA	SHRRARSVA	B0702	4.653	4721.985	BINDER	NONBINDER	1014.848
SPRRARSVA	SHRRARSVA	B4201	8.896	6168.637	BINDER	NONBINDER	693.422
SPRRARSV	SHRRARSV	B0702	24.386	12721.851	BINDER	NONBINDER	521.692
SPRRARSV	SHRRARSV	B4201	38.581	11616.598	BINDER	NONBINDER	301.097
QTNSPRRAR	QTKSHRRAR	A6801	95.531	1735.681	BINDER	NONBINDER	18.169
TQTNSPRRAR	TQTKSHRRAR	A6801	254.884	4246.926	BINDER	NONBINDER	16.662
SPRRARSVA	SHRRARSVA	B0801	372.226	3832.08	BINDER	NONBINDER	10.295
SPRRARSV	SHRRARSV	B0801	428.443	2448.483	BINDER	NONBINDER	5.715
TQTNSPRRAR	TQTKSHRRAR	A3303	195.532	659.04	BINDER	BINDER	3.37
QTNSPRRAR	QTKSHRRAR	A3303	48.633	144.583	BINDER	BINDER	2.973
QTNSPRRAR	QTKSHRRAR	A7401	3391.065	8853.904	BINDER	NONBINDER	2.611
TQTNSPRRAR	TQTKSHRRAR	A3101	144.739	336.594	BINDER	BINDER	2.326
QTNSPRRAR	QTKSHRRAR	A3101	34.812	79.999	BINDER	BINDER	2.298
SPRRARSVA	SHRRARSVA	B1402	8252.613	2833.566	NONBINDER	BINDER	0.343
SPRRARSV	SHRRARSV	B1402	13575.134	4358.653	NONBINDER	BINDER	0.321

By analysing paired HLA-specific changes in binding affinity for individual Wuhan-mutated peptides, we found substantial variation (Supplementary Fig. S2G), where some peptides, e.g. spike-derived FQPT*, FCND*, and YQP*, experienced considerable detriments to their binding capacity, while some are unchanged or perhaps exhibited stronger affinity (e.g. S:KVG*).

Our observations thus far suggest impairments in individual T cell reactivity against VOCs as observed by Naranbhai *et al.* may be conditioned by HLA genotype and bias towards certain epitopes amongst an individual’s responding T cell compartment. Indeed, by analysing the number of HLAs predicted to present each peptide before (wild type, red) and after (mutant, blue) BA.1 Omicron mutation, we found that these alterations are predicted to remove nine epitopes (9/80, ~11% of mutated CD8+ T cell targets) as predicted HLA class I ligands entirely (Fig. 2F). Although this represents a small proportion (9/1380) of the total pool of immunogenic SARS-CoV-2 CD8+ T cell epitopes, it is plausible that individuals with memory responses biased towards such epitopes may have impaired T cell responses to Omicron and possibly breakthrough infections.

Overall, we have observed that VOC mutations have the capacity to disrupt HLA binding to functionally evaluated SARS-CoV-2-specific CD8+ T cell targets. Our data suggest that the landscape of presenting HLAs is also impacted by the mutations. While Omicron mutations are only observed in small proportions of total Wuhan Hu-1 CD8+ T cell targets, given these collective data, it is plausible that patients with certain HLA and/or memory responses biased towards certain pMHC could be more affected by SARS-CoV-2 VOC mutations than the current paradigm suggests.

### Comparison of Wuhan Hu-1 and Omicron T cell immunogenicity

Peptide presentation by MHC molecules, although necessary, is insufficient to infer T-cell immunogenicity in humans [38]. To invoke a T cell response, a presented pMHC must be recognised by a cognate T cell. Therefore, to build on previous insights into HLA binding, we examined how mutations in Omicron VOCs can affect the immunogenicity potential (i.e. both HLA binding and T cell recognition) of pMHC. To do this, we combined two prediction scores for each peptide-MHC: (i) antigen presentation potential and (ii) T cell recognition potential (see Methods).

While *in silico* models that predict antigen presentation can be highly accurate (such as netMHCpan), state-of-the-art models predicting the subset of HLA ligands that then invoke T cell responses possess limited accuracy [39]. We recently developed TRAP (T cell recognition potential of HLA-I presented peptides) [33], a convolutional neural network (CNN) model that offers improved predictions of T cell recognition potential of HLA-I presented 9- and 10-mer peptides. To predict the T cell recognition potential of Wuhan Hu-1 vs. Omicron pMHC of interest, we trained an instantiation of TRAP on coronavirus epitopes from the IEDB (‘Methods’ section).

**Figure 3.**
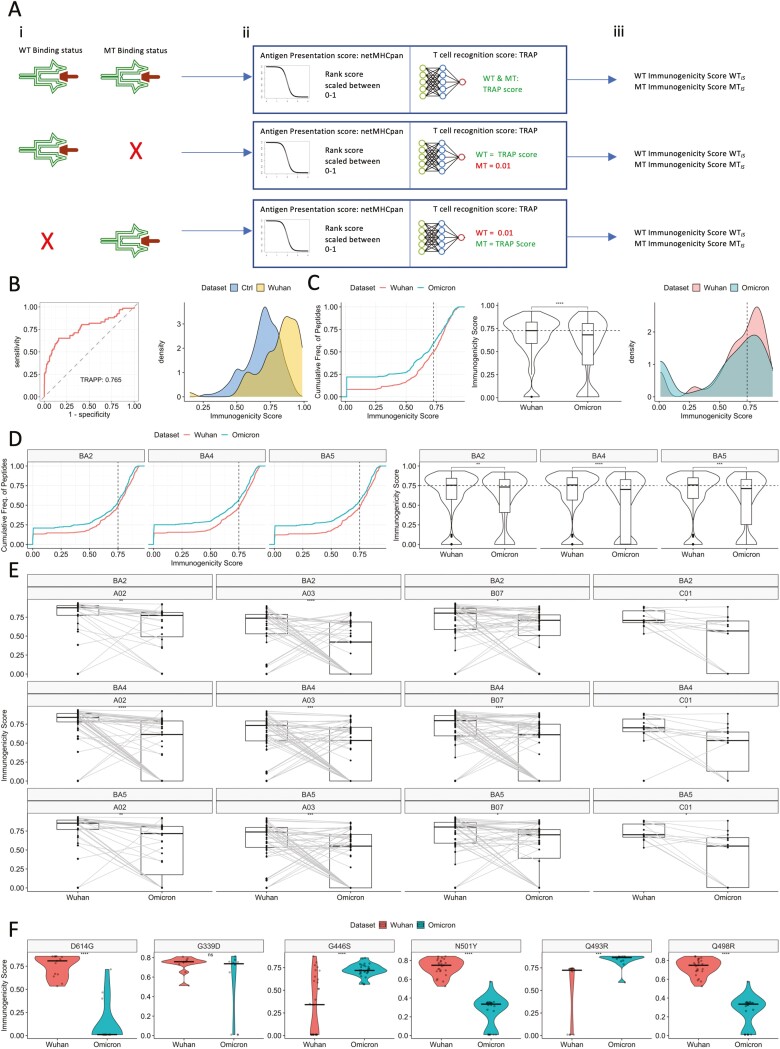
The predicted impact of BA.1 Omicron and subvariant mutations on T cell immunogenicity of SARS-CoV-2 CD8+ T cell targets. Analyses were only performed for the set of Wuhan Hu-1 CD8+ T cell targets with a mutation and their mutated counterparts (WT-MT). T cell immunogenicity incorporates (i) antigen presentation predictions by netMHCpan 4.1 and (ii) T cell recognition predictions by TRAP. (A) An overview of how the T cell immunogenicity scores were generated and the different scenarios by which imputations of pseudo-zero ‘T cell recognition’ scores (0.01) were made. TRAP by definition makes T-cell recognition predictions against peptides bound to MHC *k*. Thus, given three potential bi-directional changes in WT vs. MT binding status, three scenarios are captured. Row 1 shows a setting where for a particular CD8+ T cell target, the wildtype peptide and mutant peptide, both bind MHC *k*. Here, antigen presentation predictions were made by netMHCpan which were combined with T cell recognition scores from TRAP (see ‘Methods’ section for full details), no imputations were made. The immunogenicity score combines these two predictions. Row 2 shows a setting where the wildtype peptide binds MHC *k*, although the mutant – following Omicron mutation – does not. Here, antigen presentation is predicted using netMHCpan for both wild-type and mutant (the mutant will accordingly receive a low MHC binding score). TRAP, however, cannot make a T cell recognition prediction for a peptide not predicted to bind MHC *k*, therefore, we impute a pseudo-zero value of 0.01 for the mutant. A similar situation is depicted in row 3, however, here, the wildtype was not predicted to bind MHC *k* thus was not immunogenic, however, after Omicron mutation, antigen presentation status was observed. Here, we impute a TRAP score for the non-binding wild type and make a TRAP prediction for the presented mutant. (B) ROC curve and density plot evaluating the performance of TRAP against 66 known SARS-CoV-2 Wuhan Hu-1 CD8+ T cell targets with a mutation in Omicron (immunogenic Wuhan Hu-1 set, yellow) vs. 10 sets of 66 functionally evaluated non-immunogenic SARS-CoV-2 prediction scores made by TRAP, which were randomly sampled from a 10-fold cross-validation of training data (control, blue). ROC curves show the performance of a model through perturbing classification thresholding and visualising the true positive rate (fraction of true positives/all true positives) against the false positive rate (fraction of false positives/all true negatives). Curve information is summarised using the area under the curve (AUC). Given a balanced dataset for binary classification (50% per classification), an unskilled model will have a ROC-AUC of 0.5, reflecting only the balance in the dataset. A perfect model would have a ROC-AUC of 1.0. (C) Empirical cumulative distribution (ECDF) plot, violin plot, and density plot comparing the predicted T cell immunogenicity scores of Wuhan-mutated vs. Omicron BA.1 pMHC. Significance was assessed using a Wilcoxon rank test. (D) ECDF and violin plots comparing T cell immunogenicity scores of Wuhan-mutated pMHC vs. Omicron subvariant BA.2, BA.4, and BA.5 counterparts. Significance was assessed using Wilcoxon rank tests. (E) Paired boxplots for different HLA supertypes (HLA-A02, HLA-A03, HLA-B07, and HLA-C01), comparing the T cell immunogenicity scores between Wuhan-mutated epitopes and Omicron variant mutated counterparts. Significance was assessed using paired Wilcoxon rank tests. (F) Violin plots contrasting the Wuhan vs. BA.1 Omicron T cell immunogenicity scores for spike mutations; D614G, G446S, G339D, N501Y, Q493R, and Q498R. Significance was assessed using a Wilcoxon rank test.

To evaluate the accuracy of the trained model for our specific research question, we reserved all 66 experimentally validated 9- and 10-mer Wuhan-mutated pMHC and we compared their predicted scores with randomly sampled ‘negative’ sets 10 times (66 per repetition, Methods). We demonstrated that TRAP could identify immunogenic Wuhan-mutated pMHC with a receiver operating characteristic-area under the curve (ROC-AUC) of ~0.76 (Fig. 3B) and a precision-recall area under the curve (PR-AUC) of ~0.82 (Supplementary Fig. S3A). Therefore, we determined the model was fit for the purpose of predicting the impact of Omicron mutations on T cell recognition potential of Wuhan-mutated pMHC.

To evaluate the extent that mutation affects T cell immunogenicity, we generated ‘immunogenicity’ scores by combining (i) a ‘netMHCpan’ antigen presentation score and (ii) the ‘TRAP’ T cell recognition score (see Fig. 3A and ‘Methods’ section for complete details). By examining immunogenicity scores of 580 paired Wuhan-mutated WT vs. MT pMHC (from any protein), we observed that while global predicted T cell immunogenicity is preserved upon Omicron, BA.1 epitopes show a subtle reduction when compared with their Wuhan Hu-1 counterparts (Fig. 3C and Supplementary datafile 2 for full paired changes).

We repeated this analysis for Omicron subvariants BA.2, BA.4, and BA.5 and found the same trend (Fig. 3D and Supplementary datafile 3 for full paired changes). We also examined cross-HLA variation and observed reduced BA.1 T cell immunogenicity for HLA-A02 and -C01 ligands (Supplementary Fig. S3B). For BA.2, BA.4, and BA.5 on the other hand we observed significant impairments in T cell immunogenicity for HLA-A02, -A03, -B07, and -C01 ligands (Fig. 3E). Interestingly, the magnitude of impairment per supertype varied between subvariants, suggesting that different mutations across Omicron-based VOCs produce nuanced effects on T cell immunogenicity that appear to be HLA-dependent.

We therefore next investigated how specific point mutations affect the immunogenicity of Wuhan Hu-1 vs. Omicron pMHC (and its subvariants). Interestingly, we observed substantial variation in the effects of mutation on BA.1 T cell immunogenicity (Fig. 3F and Supplementary Fig. S3C). Indeed, mutations such as D614G (a critical Delta mutation), N501Y, and Q498R are predicted to significantly reduce T cell immunogenicity, whereas mutations such as G446S [40] and Q493R may potentiate it (Fig. 3F). In partial agreement with observations by Li *et al.* [41], our modelling predicts that G339D impairs several pMHC, although we did not observe a significant reduction in T cell immunogenicity, suggesting variation regarding how this mutation affects different pMHC. Notably, Q→R substitutions in different positions of spike protein (P493 vs. P498) are predicted to lead to opposite effects, indicating that the same amino acid change at different positions produces opposite effects on T cell immunogenicity. By examining characteristic mutations of BA.2, BA.4, or BA.5, we again observed distinct effects (Supplementary Fig. S3D).

Lastly, we integrated our data with metadata of Naranbhai *et al.*’s patients who exhibited impaired T cell responses. We found that for 6/10 patients, ≥40% of their carried HLAs were associated with reductions in predicted T cell immunogenicity given BA.1-mutated epitopes, while for two patients, ≥60% of HLAs are impacted (Supplementary Fig. S3E). Although we cannot draw a firm conclusion due to the absence of control data, these findings build on the insight of Naranbhai *et al.*, who argued that the HLA genotype of these patients may in part contribute to the impairments observed in their study.

Overall, our data indicate that while broadly the T cell response is preserved following Omicron mutations, their impact on the landscape of T cell immunogenicity is divergent and heterogeneous. This work provides insights into how different mutations are likely to affect T cell immunogenicity.

### Relative impact of Omicron mutation on potential and breadth of T cell responses

We have observed that Omicron mutations have disparate effects on pMHC immunogenicity. In general, mutation(s) at a CD8+ T cell target may impact the overall potential (i.e. change in likelihood of T cell response) and breadth (change in the number of cognate T cells in a given repertoire). To explore this further, we set out to (i) profile the relative impact of Omicron mutation on likelihood of T cell immunogenicity (potential) for each pMHC (with respect to Wuhan Hu-1) and (ii) estimate the effect of mutation on promiscuity of binding cognate T cell receptors (TCRs) (breadth). To achieve the first goal, we computed a metric which we termed ‘relative immunogenic potential’ (RI) (‘Methods’ section). RI is computed for each *pMHC* and a score of >0 indicates that the Omicron mutation(s) potentiates immunogenicity, while <0 indicates impairment.

At the same time, we aimed to estimate the effects of BA.1 Omicron mutations on response breadth and whether such mutations may trigger broad, promiscuous T-cell activation. As Omicron and its subvariants spread globally, we predicted whether Omicron mutations may alter the breadth of TCRs recognising mutated peptides.

We thus retrained TITAN [42], a state-of-the-art CNN model for computational mapping of TCRs to their target antigens (see ‘Methods’ section and Supplementary Methods). Therefore, TITAN allows interrogation of the breadth of T cell responses to a given epitope. After observing reasonable performance (ROC-AUC 0.74 albeit with considerable variation across epitopes), we took our unseen TCR dataset (162,930 TCRs, Supplementary Methods) and predicted their binding against each (unseen) Wuhan-mutated or counterpart Omicron peptides. TITAN does not consider HLA, thus, for each *peptide*, we calculated RTP: ‘relative TCR promiscuity (Methods)’, comparing the number of TCRs predicted to bind the Wuhan Hu-1 WT vs. Omicron MT. RTP > 0 reflects increased breadth after Omicron mutation; simply, binding more TCRs.

**Figure 4.**
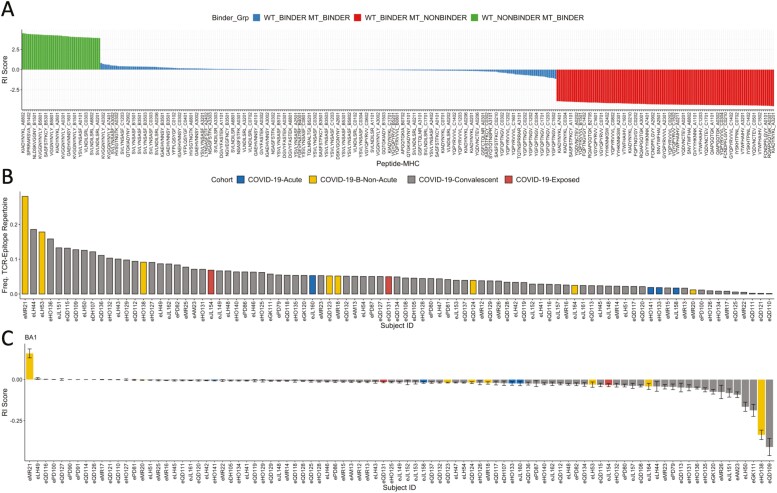
The overall impact of Omicron mutations on immunogenic potential of pMHC and individual TCR repertoires. (A) Barplots showing the relative immunogenic potential (RI) score for each pMHC affected by a mutation in BA.1 Omicron. Labels are truncated for visual clarity. RI scores for each pMHC are supplied in Supplementary datafile 3. Colour labelling represents groups of pMHC by wildtype←→ mutant binding status: WT_BINDER_MT_BINDER (blue), WT_BINDER_MT_NONBINDER (red), and WT_NONBINDER_MT_BINDER (green), representing, respectively, pMHC where both the WT and MT bind the same MHC (blue), the WT binds a particular MHC but the mutant does not (red), the WT does not bind a particular MHC that the mutant does (green). RI is produced for each pMHC. (B) Barplot showing the frequency of individual MIRA TCR-epitope repertoires (excluding healthy individuals) which exhibit a mutation in BA.1 Omicron. (C) Barplot showing the mean ± standard error for ‘RI’ scores for each individual MIRA TCR-epitope repertoire. BA.1 only. As MIRA antigen-specific data do not explicitly label the bound MHC to the antigen, RI scores here are computed for each peptide, thus represent the mean RI across predicted MHC (pan-HLA RI). Scores of ‘zero’ are imputed for non-mutated epitopes, thus representing no change in immunogenicity between Wuhan Hu-1 and BA1 Omicron. Visually, ‘pan HLA’ RI scores are summarised here for each patient.

By analysing both ‘RI’ and ‘RTP’ (Supplementary datafile 4 for full dataset), we found a divergent landscape for the impact of BA.1 mutations on immunogenic potential (i.e. RI, Fig. 4A) but no pattern for breadth (Supplementary Fig. S4A). There are small to moderate impairments (RI < 0) in potential for a substantial number of pMHC, while others are eliminated as CD8+ T cell targets through inability to bind MHC. In contrast, there are pMHC with enhanced potential following the mutation in Omicron.

While a handful of peptides that compose pMHC with enhanced potential are predicted to additionally bind more TCRs (Supplementary Fig. S4A/Fig. 4A), we were unable to draw meaningful conclusions regarding whether mutations impacted TCR breadth. Nevertheless, we propose that these pMHC (e.g. ‘GYQPYRVVVL_B1402’, ‘LYNSASFSTF_B5201’) with >0 RI score (enhanced potential) which are composed of peptides with predicted RTP > 0 (increased breadth), may be of interest for investigations into post-COVID-19 inflammatory disorders.

Thus far, our predictions show that mutations in Omicron BA.1 (and its subvariants: Supplementary Fig. S4B) produce heterogeneous effects on the immunogenic potential of pMHC, including some complexes that are removed as CD8+ T cell targets (e.g. CNDF*, see Fig. 2F). Furthermore, we have observed that peptides bound to certain HLAs (e.g. A03, A02, and B07) are associated with a reduction in immunogenicity, while other HLAs are not. Collectively, this heterogeneous impact implies that individuals with certain HLAs and/or TCR repertoires biased towards certain epitopes will be more affected than others.

We thus sought to expand this approach to investigate the impact of mutations on antigen-specific T cell immunogenicity potential, at the level of individual TCR repertoires. We, therefore, extracted 160k high-confidence SARS-CoV-2-specific TCRs from 93 COVID-19 patients (79 convalescent + 14 acute/non-acute/exposed) from the ‘MIRA’ dataset [43]. The data consist of 543 Wuhan Hu-1 SARS-CoV-2 peptides, 46 (~8%) of which are mutated in Omicron. These individuals’ TCR repertoires were subjected to our RI score (see ‘Methods’ section).

We, therefore, treated each resulting TCR-epitope repertoire as a representation of Wuhan Hu-1-induced memory and interrogated how Omicron mutations may affect overall potential of T cell response. First, for each subject we calculated the proportion of their TCR-epitope repertoire which targets Wuhan Hu-1 epitopes which exhibit a mutation in BA.1 Omicron (Fig. 4B). Consistent with our previous observations, we observed a variety of different impacts, ranging from <1% to >20%. We next analysed the overall effects given the average RI scores for each individual’s antigen-specific TCR repertoire (see ‘Methods’ section).

We found that while many individuals were likely to exhibit no effect given overall RI scores, a set of individuals showed impairments (Fig. 4C and Supplementary Fig. S4C). We did not find any association between these impaired individuals and available clinical metadata or, e.g. size of the responding repertoires (Supplementary Fig. S4D). It is plausible that reduced immunogenic potential that impacts overall T cell response, may in part contribute to breakthrough infection, although further investigation is clearly required. Nevertheless, our data support the hypothesis that an underlying cause of the heterogeneity observed with T cell impairment, given VOC mutations, is due to biased responses towards certain epitopes exhibiting impairments after mutation, which may be conditioned by the HLA genotype.

### Inference of SARS-CoV-2 T cell escape potential by *in silico* mutagenesis

The set of VOC mutations that affect CD8+ T cell epitopes are a subset of theoretical mutational space; however, the underlying sampling mechanism is unclear. Sampling under some form of evolutionary pressure may suggest that the virus is taking a path to escape cellular immunity. Indeed, as optimal ACE2 affinity and cell entry are approached, wider forms of escape are likely to be evaluated by the virus [32]. A first step towards addressing this fundamental question and estimating the impact of emergent mutations on T cell responses is to evaluate the impact of each theoretical single-point mutation in a CD8+ T cell epitope on its immunogenicity.

To address this, we combined *in silico* mutagenesis and our immunogenicity modelling. As a proof of concept, we used the BA.1 Wuhan-mutated 9/10-mer epitopes as a model system. For each Wuhan-mutated wild-type peptide (*n* = 66) (WT_i_), we generated each theoretical mutant (MT_*k* = 1…*n*_, *n* = 171 for 9-mers and 190 for 10-mers, see ‘Methods’ section). We then produced immunogenicity scores for predicted peptide-MHC as described previously, which we subsequently analysed in a pan-HLA manner (see Fig. 3A and ‘Methods’ section).

**Figure 5.**
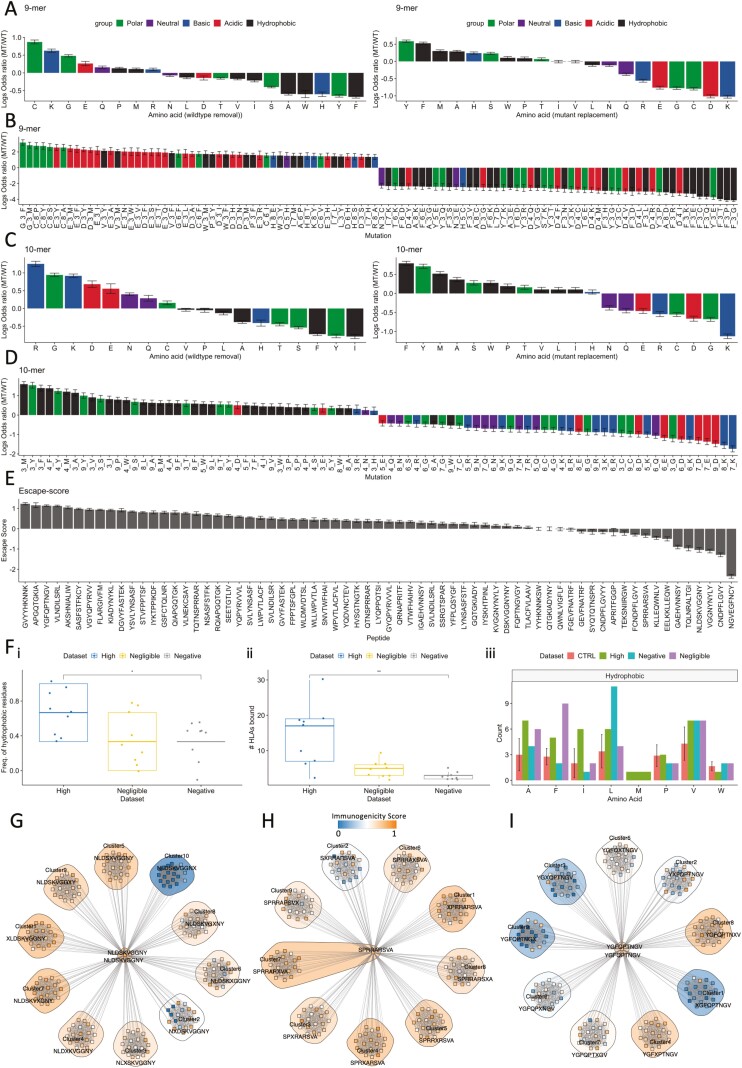
The effects of theoretical mutations on SARS-CoV-2-specific CD8+ T cell epitopes. (A–D) Barplots showing the mean ± standard error of log ratios derived from comparing mutant/wildtype immunogenicity scores from simulated mutations in TCR contact positions of (A and B) 9-mers and (C and D) 10-mers. Negative log ratios demonstrate that the mutant is less immunogenic than the wild type and vice versa. (A) Barplot showing for each amino acid residue, the mean ± standard error across of all log ratios (9-mers). Left plot shows the log ratios upon removing a particular amino acid from the wildtype sequence. Right plot shows the log ratios upon imputing a particular amino acid in the mutant. Colour labels show chemistry of amino acids. (B) The highest and lowest 5% of log ratios derived by analysing the full mutations (A_*x*_B) simulated in TCR contact positions of 9-mers. Colour label shows the chemistry of the removed amino acid. (C) Barplot showing for each amino acid residue, the mean ± standard error across all log ratios (10-mers). Left plot shows the log ratios derived from removing a particular amino acid from the wildtype sequence. Right plot shows the log ratios derived from imputing a particular amino acid into the mutant. Colour labels show chemistry of amino acids. (D) Highest and lowest 30% of log ratios showing impact on immunogenicity for replacing residues (i.e. those amino acids inserted) into position *x* of TCR contact positions of 10-mers. (E) Barplot showing ‘escape scores’: the mean ± standard error of the difference between WT and MT (WT minus MT) immunogenicity scores affecting each assessed SARS-CoV-2 CD8+ T cell target (see Methods for full details). (F) (i) Boxplot comparing the proportion of hydrophobic residues in non-TCR contact positions of three groups of peptides (*n* = 9 per group, containing both 9- and 10-mers): (blue) ‘high’, (yellow) ‘negligible’, and (grey) ‘negative’, grouping epitopes with (blue) highest escape score, (yellow) escape score approx. 0, and (grey) negative escape scores, respectively. Nine samples were chosen to maximise samples whilst comparing the same quantity of peptides across groups. Significance was assessed using a Wilcoxon rank test. (ii) Boxplots comparing the number of HLAs predicted to bind the nine selected wildtype peptides for each ‘escape score’ group of interest. (iii) Barplot showing the number of times each hydrophobic amino acid is observed in wildtype peptides composing the three ‘escape’ groups. A control group (CTRL, red) was generated to assess significance. The control group represents a bootstrapped background distribution of counts of hydrophobic amino acids from sampling nine peptides randomly from the entire distribution of Wuhan-mutated 9- and 10-mer peptides. Crossbars show standard error. (G–I) Neighbour-network diagrams depicting the impact of mutation via network trajectories from the wildtype peptide (centre) to each single amino acid variant. Clusters and individual mutants are colour labelled by immunogenicity score. Groups are clustered by location of the substitution. An *x* in the consensus sequence by the cluster indicates the position of the substitution that characterises the cluster.

Next, we analysed log ratios between each MT_k_ and its corresponding WT_i_ at TCR contact positions of 9-mers. Focusing on each amino acid (and not position), we found that the removal of polar residues cysteine (C), glycine (G), and basic lysine (K) from wildtype peptides increased immunogenicity (log ratio > 0) (Fig. 5A – left, Supplementary Fig. S5A). Similarly, by incorporating these residues in the mutant (i.e. replacement), immunogenicity was reduced (Fig. 5A – right, Supplementary Fig. S5A). On the other hand, in most cases (6/8) the removal of hydrophobic residues (tryptophan (W), phenylalanine (F), alanine (A), etc.) in TCR contact positions impaired immunogenicity. Next, we examined the impact of full mutations (A_*x*_B: A = removal, *x* = sequence position, B = replacement) occurring in the TCR contact positions of 9-mers. Focusing on the mutations with the highest and lowest 5% of logs ratios, we again observed that primarily, the removal of hydrophobic residues impaired immunogenicity (Fig. 5B). Interestingly, mutations that removed a basic amino acid predominately potentiated immunogenicity, while removals of phenylalanine (F) or tyrosine (Y) from P3 were commonly found amongst the most detrimental. Similarly, by examining TCR contact positions of 10-mers, removals of arginine (R), glycine (G), and lysine (K) potentiated immunogenicity, whereas removals of hydrophobic residues were again detrimental (Fig. 5C and Supplementary Fig. S5B).

Interestingly for 10-mers in TCR contact positions, when we examined the impact of the *replacing* amino acid in position *x* we found a skew in underlying chemistry associated with immunogenicity enhancements or detriments (Fig. 5D). In fact, hydrophobic and polar residues dominated the most immunogenic replacements, while detrimental replacements comprised more of a balance of acidic, basic, and neutral residues. These findings not only provide a viewpoint into the molecular biology that underpins our immunogenicity modelling but also suggest that physicochemical properties of amino acids (wildtype and/or replacement) may be critical for estimating the impact of mutation on T cell immunogenicity and contribute to how escape-prone a specific epitope is.

To identify the most and least escape-prone CD8+ T cell targets given theoretical mutations, we defined ‘escape score’ (‘Methods’ section), as average log ratio changes across all mutants for each epitope of interest (Fig. 5E, Supplementary datafile 5). We found varying degrees of overall impact associated with three groups: (i) those predicted with *high* escape scores, e.g. spike-derived GVY* and YGF* showing overall impairment to immunogenicity, (ii) those predicted with *negligible* escape scores, thus are tolerant to mutation (mean ± standard error approx. 0), e.g. membrane-glycoprotein TLAC*, spike-derived YYH*, and (iii) those with a *negative* ‘escape score’, e.g. NGV*, spike-derived NLD*, which are on the other hand predicted with global improvements to their immunogenicity as a result of mutation.

Given the associations between removal of hydrophobic residues and impaired immunogenicity, we explored whether properties of wildtype peptides may contribute to ‘escape score’. We found that escape-prone wildtype peptides (e.g. spike-derived APG*) have a slightly higher proportion of hydrophobic residues in non-TCR contact positions (1, 2, and 9) of 9-mers compared to those peptides where mutation potentiated immunogenicity (e.g. spike-derived NGV*) (Fig. 5F-i). Additionally, we found that escape-prone epitopes bind more HLA (Fig. 5F-ii), which may increase opportunities for escape. Furthermore, wild-type epitopes containing phenylalanine (F) seem to be more tolerant to mutation (Fig. 5F-iii). These data suggest that while escape potential is multifaceted, the hydrophobic complexion and HLA promiscuity of the wild type may guide the likelihood and direction that mutation impacts its immunogenicity.

Next, to visualise in detail the predicted mutational trajectories of each epitope, we implemented the neighbour-network strategy from Ogishi *et al.* [44] (Methods).

Through theoretical mutations, NLD* was predicted with overall improvements to its immunogenicity, thus a *negative* escape score (see Fig. 5E, right). This epitope is already highly immunogenic; therefore, our analysis indicates that this epitope is a robust target, while on average mutations enhanced immunogenicity (Fig. 5G). Notably, this epitope could tolerate many mutations in the P2 anchor position, whereas mutations in the P10 anchor position eliminated immunogenicity. Given this epitope’s general tolerance to mutation, it may be of interest, e.g. for T cell therapies.

Spike epitope SPR* should be robustly immunogenic given our predictions (Fig. 5H). Only mutations in P2 considerably impaired immunogenicity. With Omicron, this epitope is affected by a P→H mutation in P2, which drastically compromised binding to HLA-B07 alleles. These data indicate that in the ‘real world’ – through VOCs – SPR* has taken one of the few available escape routes. Our analysis showed that after mutations in VOCs, SPR* will possess a narrower MHC binding repertoire. It is, therefore, plausible that an escape route has been taken – perhaps due to a highly immunogenic Wuhan Hu-1 wildtype – leaving only a single, highly potent pMHC (HLA-B*14:02-SPR*).

Our analysis predicts a high escape score for spike-derived YGF* (Fig. 5E, left) via substantial numbers of escape routes (Fig. 5I). These data suggest that most mutations in anchor positions P1 (cluster 1) and P9 (cluster 9), as well as TCR contacting P3 (cluster 3) will damage immunogenicity. YGF* is affected by three Omicron mutations: G496S, Q498R, and N501Y affecting P2, P4, and P7 of the epitope, respectively. Our immunogenicity modelling predicts that across each bound MHC, Omicron causes low immunogenicity for YGF* (immunogenicity scores 0.26–0.34, Supplementary datafile 1). Indeed, the predicted effects of Omicron’s impact given three aggregate mutations may be unsurprising, as the neighbour-network analysis indicates that YGF* was highly susceptible even to single-point mutations.

Overall, this analysis highlights the utility of applying *in silico* mutagenesis and immunogenicity modelling to examine the effect of theoretical mutations on T cell responses. We have observed varied effects of single-point mutations on the immunogenicity of different epitopes. The ability to predict the impact of mutations on T cell immunogenicity would provide a powerful way to quickly understand the impact of an emergent VOC on a critical arm of the immune response. In a small step in this direction, we have developed an R script which, for a given mutation, amino acid removal or replacement, provides an estimate of the effect on T cell immunogenicity, given our *in silico* mutagenesis data. This work, therefore, provides a gateway towards a strategy that given further validation and data, has the potential to reduce uncertainty associated with emergent VOCs and their impact on T cell responses.

## Discussion

The threat of a future VOC characterised by T cell evasion is an ongoing concern. As SARS-CoV-2 approaches optimal cell entry, other selection advantages, e.g. T cell escape, may be utilised to increase pathogenic fitness [32]. While extensive research has been undertaken to estimate effects of SARS-CoV-2 mutations on antibody recognition, mutational impact on T cell epitopes is poorly understood. Indeed, the ability to rapidly estimate the extent to which mutations impact T cell immunogenicity would reduce uncertainty upon emergence of novel VOCs.

In this study, we have used *in silico* approaches (i) to examine how mutations among Omicron VOCs affect SARS-CoV-2-specific CD8+ T cell responses and (ii) to provide a preliminary framework for how theoretical mutations can impact SARS-CoV-2 epitopes. Our study is a step towards estimating the impact of mutation(s) on T cell immunogenicity upon the emergence of novel VOCs. We demonstrate how T cell immunogenicity modelling can be used to not only dissect the impact of *existing* mutations on T cell responses but also to forecast *theoretical* mutations that may be most detrimental for T cell immunity when combined with *in silico* mutagenesis. We envision that this work can support ongoing surveillance and forecasting efforts [45–47] and serve as a foundation of a knowledge base for determining how VOCs may impact T cell responses upon their emergence. As model performance improves and more data are generated, the uncertainty regarding how emergent VOCs may affect T cell immunity could be reduced.

As Dolton *et al.* recently highlighted, ‘broad brush’ approaches have failed to uncover evidence of T cell escape following key variant mutations [10, 32]. Indeed, during the early stages of Omicron’s rise to global dominance, the overwhelming consensus argued that Omicron does not significantly impair T cell responses [7, 10, 48–53]. Due to the diversity of HLA and breadth of epitopes, it had rightly been reasoned that widespread and complete T cell escape is unlikely at this stage of the pandemic [16, 54–56].

In that light, *in silico* studies e.g., the work by Nersisyan *et al.* [29] compared all theoretical HLA ligands across specific VOCs and concluded that T cell responses to Omicron were likely to be maintained effectively. However, not all HLA ligands can invoke T cell responses [38, 39]. Furthermore, studies such as those of Naranbhai *et al.* [17] and Reynolds *et al.* [18] observed considerable numbers of patients with impaired T cell responses to Omicron infection [57]. Indeed, while widespread and complete escape is unlikely, it is plausible that detrimental mutations could impair certain individuals, e.g. with certain HLAs and narrow TCR repertoires targeting impacted pMHC. Thus, we hypothesised that some Omicron mutations may be more detrimental than the consensus, affecting the responses of some individuals more than others. This hypothesis was supported by *in silico* work by Pretti *et al.* [31] indicating heterogeneous effects on binding HLA, given mutations among VOCs prior to Omicron.

We thus investigated CD8+ T cell targets with a mutation in Omicron and used predictive modelling to infer the effects of each mutation on T cell immunogenicity. Our work supports a body of literature that indicates overall, there exists a subtle reduction in T cell immunogenicity with mutated epitopes in Omicron VOCs, compared to Wuhan Hu-1. By examining mutated epitopes in detail, our study extends this paradigm, revealing a divergent landscape for how different CD8+ T cell targets are affected by Omicron-based mutation(s). In support of our hypothesis, we found that this heterogeneous landscape may produce a net impairment on some individuals’ T cell response against Omicron VOCs, likely conditioned by a bias towards certain epitopes in individual repertoires and patient HLA genotype. We speculate that these combined factors may lead to T cell escape in some individuals and perhaps breakthrough infections, although – as many epitopes are not mutated in VOCs – the extent to which mutated epitopes contribute to clinical outcome remains to be elucidated. Additionally, while our modelling analysed Omicron mutations in Wuhan Hu-1-derived epitopes, which we hypothesise may impact breakthrough infections, novel immunogenic epitopes in different regions of SARS-CoV-2 may have been generated via Omicron. Therefore, investigations into such epitopes would be required to understand how Omicron infection may affect naïve individuals, compared with breakthrough infections in individuals who possess natural or acquired immunity to Wuhan Hu-1.

Evidence of SARS-CoV-2-specific T cell escape has begun to emerge. For example, Stavenich *et al.* [58] identified two mutations in a non-Hodgkin’s lymphoma patient infected with SARS-CoV-2, which escaped CD8+ T cell responses. Recently, Dolton *et al.* focused on the dominant HLA-A02 spike epitope YLQ, which was found to evade >175 TCRs due to a P→L mutation. Before Omicron’s emergence, Hamelin *et al.* [34]. found that proline removals in Delta and Alpha could damage HLA-B07 pMHC binding. Extending these insights, our modelling predicted that P→X Omicron mutations eliminate certain HLA-B07 pMHC. Notably, subsequent to our predictions and independent of our study, Swaminathan *et al.* have shown that CD8+ T cells fail to recognise the Omicron-mutated HLA-B07:02 ligand SHR*, while SPR* was recognised. This confirms a prediction made by our study, that the P→H mutation in P2 of this epitope would eliminate immunogenicity (see Fig. 2D). Our work, combined with Hamelin, Dolton’s and Swaminathan’s indicates that P→X mutations – which are now observed across Omicron variants that have infected large proportions of populations – in CD8+ T cell epitopes should be a priority for surveillance regarding T cell escape in HLA-B07 individuals and for associations with breakthrough infections. Overall, our data combined with recent studies are starting to indicate a more nuanced outlook regarding the impact of VOCs mutation on T cell responses, than the consensus [17, 31, 32, 34, 57, 59].

There are ongoing efforts to characterise disorders that are considered ‘post-COVID’ complications, ranging from so-called long-COVID [21, 22, 60–63] to broad inflammatory disease [20, 23–25]. A controversial hypothesis regarding observations of multisystem inflammatory disorder is that a theoretical SARS-CoV-2 superantigen promotes aberrant T cell activation [20, 23]. The ‘PRRA’ motif, part of this hypothesised core of a SARS-CoV-2 superantigen [26, 27] overlays three CD8+ T cell epitopes that have mutations in BA.1 Omicron. Our data indicate that after Omicron mutation, 9/15 of the pMHC containing this theoretical superantigen core are removed as HLA ligands. If functional evidence is attained for this SARS-CoV-2 superantigen, we propose that remaining presenters HLA-A*33:03, -A31:03, and -B14:02 be investigated for associations with inflammatory disorders following COVID-19 infection. More generally, we have predicted that a set of pMHC is likely to exhibit increased immunogenicity and bind more TCRs following Omicron mutation. Overall, further investigation is warranted to assess these particular CD8+ T cell targets, HLAs, and cognate TCRs, for associations with post-COVID inflammatory disorders.

With potential exceptions (SPR*, Fig. 5H), our data do not suggest that reductions in T cell immunogenicity observed with Omicron were the result of immune pressure. Nevertheless, many evaluated epitopes were predicted to be escape-prone, albeit with variation, given theoretical single-point substitution. This insight could be concerning, given the considerable number of unexplored mutations available for SARS-CoV-2 that our data estimate could lead to T cell escape. Notwithstanding, mutations are not uniformly generated; thus, future work should seek to understand the likelihood of each potentially problematic theoretical mutation, perhaps by simulating evolution, e.g. using SANTA-SIM [64] or forecasting driver mutations [47].

While our study focused on Omicron lineages due to the high quantity of mutations with respect to Wuhan Hu-1, variants such as Delta played a significant role during the pandemic, thus we explored the effect of Delta mutations on immunogenicity. We observed that Delta produced a significant reduction in T cell immunogenicity among mutated epitopes compared with Wuhan Hu-1 (Supplementary Fig. S6A). Furthermore, *in silico* mutagenesis predictions identified one highly escape-prone epitope (Supplementary Fig. S6B). Nevertheless, due to a small sample size for Delta (22 epitopes exhibit mutations), we focused our investigations on Omicron lineages.

Our approach may have a limitation in calculating RI scores, where we modelled antigen presentation in a binary manner and imputed pseudo-zero T cell recognition scores for non-binders, which were included in downstream analysis. Here, if a WT peptide binds MHC *k* while the mutant does not, the immunogenicity equation (product of antigen presentation and T cell recognition) for the mutant is skewed to values close to zero and vice versa. This causes a large effect on RI scores (Fig. 4A). Modelling antigen presentation in this manner can be justified by its binary nature, thus we applied a negative logistic function to generate values approaching 1 for strong binders and 0 for non-binders. Furthermore, non-binders cannot canonically be recognised by T cells; thus, a pseudo-zero score for T cell recognition is logical.

It is of great interest to forecast future VOCs and/or determine whether Omicron emerged under a level of T cell pressure. While we had initially aimed to explore these fundamental questions, due to data limitations and that immune pressure has generally been attributed to antibody responses, we are unable to draw conclusions regarding the evolution of Omicron-based VOCs. Nevertheless, future work should examine evolution through Beta, Delta, and Omicron variants to evaluate how these developed with aims of leveraging this information to forecast future VOCs.

Another limitation is that we are unable to directly analyse the effects of mutations in T cell epitopes on breakthrough infections. This is due to the lack of publicly available data to support this analysis, which would require antigen-specific memory TCR repertoires linked with clinical information prior to and after Omicron infection. It will be important to examine whether patients who experienced breakthrough infections are enriched for T cell responses that are dominant towards epitopes we have predicted will exhibit a reduction or loss of immunogenicity after Omicron. This work would help us understand to what extent the loss of immunogenic epitopes due to mutation affects clinical outcomes.

In conclusion, we have utilised *in silico* approaches to assess the impact of existing and theoretical mutations on SARS-CoV-2 CD8+ T cell targets. We reveal a divergent, heterogeneous landscape of impact for Omicron VOCs. We proposed a framework for forecasting the effects of theoretical SARS-CoV-2 mutations using immunogenicity modelling and *in silico* mutagenesis. We hope that our work provides a gateway towards a comprehensive and publicly available approach for assessing the impact of mutations on T cell immunogenicity upon emergence of novel VOCs. In the case of detrimental impacts, we envision this approach, given further development, could rapidly generate a profile of affected epitopes and HLAs to estimate populations that are most likely be affected by novel VOCs. Our framework should next be applied to all known SARS-CoV-2 CD8+ T cell epitopes to (i) fully characterise escape-prone vs. robust epitopes, (ii) identify further theoretical mutations warranting surveillance, and (iii) further molecular insight into the biology underpinning mutation impact.

## Methods

### Data analysis

Data processing and analysis were performed with R or Python 3.7. Visualisations were made using the R library *ggplot2* or *ggpubr*.

### Curating immunogenic SARS-CoV-2 CD8+ T cell targets

A total of 1406 unique immunogenic MHC-I SARS-CoV-2 epitope data evaluated in the context of humans were gathered from IEDB and MIRA. Of these epitopes, 26 were excluded due to their origin in a non-ancestral strain. In total, 1380 unique SARS-CoV-2 Wuhan Hu-1 immunogenic epitopes were selected for analysis.

### Retrieval of SARS-CoV-2 proteomes

The Wuhan Hu-1 proteome was obtained from: https://www.ncbi.nlm.nih.gov/nuccore/nc_045512.2

Representative strains for the proteomes of Omicron BA.1, BA.2, BA.4, and BA.5 were retrieved from BV-BRC: the SARS-CoV-2 Variants and Lineages of Concern resource:

https://www.bv-brc.org/view/VariantLineage/#view_tab=overview.

### MHC presentation prediction

Antigen presentation by MHC class I was predicted using NetMHCpan v4.1 against 64 HLA types. These HLA are listed in Supplementary Methods. Peptide-MHCs with a binding affinity rank score ≤2.0 were classified as binders.

### Immunogenic Wuhan Hu-1 epitopes with a mutation in Omicron and its subvariants

For each Wuhan Hu-1 epitope, we performed a local-global alignment using *pairwiseAlignment* function from the R package *Biostrings* against each protein in Omicron proteomes. The maximum alignment across the proteome was selected as the counterpart variant. Mutation information was recorded and mapped with a known list of mutations of the respective variant, downloaded from https://covariants.org/ and https://github.com/hodcroftlab/covariants.

### Mutation effect on antigen presentation

Paired WT-MT samples were analysed by comparing predicted binding affinity nM, netMHCpan rank and the ratio of mutant/wild-type predicted binding affinity in nM (agretopicity). We focused on samples where *either* or *both* the WT and MT were predicted to bind a particular allele. The reasoning for this was to incorporate the following three scenarios: (i) where the WT is predicted to bind but the MT is not, (ii) where the WT is not predicted to bind MHC but after mutation, the mutant becomes a binder, and (iii) where both the WT and MT bind a particular MHC.

### Training TRAP to predict T cell recognition of SARS-CoV-2 epitopes

Immunogenic and non-immunogenic peptides of lengths 9 and 10 were retrieved from the IEDB in July 2022. Peptide-MHC samples from antigen organisms containing the words ‘coronavirus’ or ‘SARS’ were retained. Binary ‘positive’ or ‘negative’ labels were extracted. 37 peptides with 1 immunogenic but >2 non-immunogenic observations were excluded as this suggests the immunogenic observation could not be replicated. Next, we confirmed that all epitopes only contain amino acid characters. We excluded any pMHC that did not have four-digit resolution HLA information. These filters left 1641 distinct pMHC for analysis; 566 (34.5%) were non-immunogenic, 1075 (65.5%) were immunogenic.

For any remaining contradictory pMHC (those with both immunogenic and non-immunogenic observation(s)), we assumed these epitopes can be immunogenic thus we excluded the ‘negative’ observations. To avoid overfitting, we excluded any pMHC which contained Wuhan Hu-1 epitopes with a mutation in Omicron. We, therefore, used 1511 coronavirus epitopes to train the coronavirus TRAP model, 1074 (~71%) were immunogenic, 437 (~29%) non-immunogenic.

### TRAP model architecture

TRAP is a 1D CNN using ProtT5-XL-UniRef embeddings to predict the CD8+ T cell recognition potential of HLA-I ligands from peptide sequences. To address data limitations we utilised transfer learning, thus encoding properties of amino acids derived from protein transformed-based pre-trained language models (PLMs). These protein transformer-based PLMs have been pre-trained using millions/billions of protein sequences and carry representative 1024 embeddings that describe the physicochemical, structural, or electrostatic amino acid properties.

Predicted HLA-I ligands are required for input. TRAP then uses a 1D CNN with kernel sizes 1, 3, 5, and 7 to extract T cell recognition motifs, which are then combined with the MHC binding rank score predicted by netMHCpan and the proportion of hydrophobic amino acids to predict immunogenicity. TRAP includes a decision tree classifier to detect low-confidence predictions to improve accuracy. Hyperparameters of the model were optimised by grid search. TRAP employs different modes for (i) pathogenic ligands and (ii) self-antigens. For the pathogenic model (used in this article), the final hyperparameters are: learning rate = 1e-05, weight decay = 1e-06, dropout rate = 0.1, batch size = 50, dense layer node = 2000, and dense layer node = 256.

### Evaluating TRAP’s performance

A total of 66 functionally validated pMHC-containing peptides with a mutation in Omicron were excluded from training and reserved for testing. To generate a ‘negative’ set, 66 predictions for non-immunogenic pMHC were sampled 10 times from a 10-fold cross-validation of the training data. At each sampling, ROC-AUC, PR-AUC, precision, recall, sensitivity, specificity, balanced accuracy, detection rate and prevalence, F1, and negative/positive predictive values were evaluated. For ROC-AUC and PR-AUC plots, the data across ten samplings were summarised.

### Comparing T cell immunogenicity between Wuhan Hu-1 and Omicron variants

We compared overall predicted T cell immunogenicity of Wuhan-mutated epitopes and their mutant counterparts. We reasoned that ‘overall’ T cell immunogenicity encompasses two components: (i) antigen presentation and (ii) T cell recognition, where antigen presentation is a prerequisite for T cell recognition. We combined two metrics for (i) antigen presentation and (ii) T cell recognition to generate an ‘overall’ immunogenicity score.

For antigen presentation, we generated netMHCpan percentile rank binding-affinity estimates for each peptide-MHC. As different HLA bind their ligands in different nM ranges, percentile rank was used to reduce bias across HLA alleles. Percentile rank was transformed between 0 and 1 by use of a negative logistic function:


$$\begin{array}{l} MHC\;score(x)=\frac{1}{1+e^{k}(x-2)} \end{array}$$

The function provides a value approaching 0 for a percentile rank much higher than 2.0, the recommended threshold for discriminating netMHCpan binding. Where percentile rank = *x* = 2, the function evaluates to 0.5. For very low percentile ranks i.e., strong binders, the function gives a value approaching 1. *k* is a parameter which defines the shape of the curve. We selected *k* = 1.5, to avoid too strong skews on either side of the midpoint percentile ranks. A range of values for *k* was explored with no change to conclusions. ‘MHCScore’ was generated for each WT and each MT pMHC of interest.

We generated a ‘T cell recognition’ score for each WT and each MT pMHC using TRAP. As TRAP predicts T cell recognition, it naturally only considers samples in which binding to MHC is achieved. However, to address changes between WT and MT viral strains fairly, one must account for three groups of WT-MT MHC binding relationships: (i) samples where WT was predicted to bind MHC-*i*, but MT does not, (ii) samples where WT and MT are both predicted to bind MHC-*i*, (iii) samples where the WT was not predicted to bind MHC-*i* but MT is now predicted to bind. Samples where both WT and MT were not predicted to bind MHC were discarded.

In scenarios 1 and 3, TRAP cannot produce a score for both WT and MT pMHC, as either the WT or MT is not predicted to bind MHC-*i*. Therefore, for predicted non-binders, we imputed a pseudo-zero ‘T cell recognition’ score of 0.01, as by definition non-binders cannot be recognised by T cells. Any binders were subjected to TRAP to generate a ‘T cell recognition’ score.

The ‘overall’ immunogenicity score for each pMHC of interest, is given by:


$$\begin{array}{l} ImmunogenicityScore\;=\;MHCScore\;\;\;\times \;\;TRAPscore \end{array}$$

Samples in which both WT pMHC and MT pMHC are predicted with immunogenicity scores <0.5 were excluded, as we conservatively estimate that these are not immunogenic and therefore are not of interest.

### Relative immunogenic potential (RI score)

For a pMHC of interest:


$$\begin{array}{l} RI=log\left(\frac{MT\_Immuno}{WT\_Immuno}\right) \end{array}$$

where *MT_Immuno* and *WT_Immuno* are the immunogenicity scores for the MT pMHC and the WT pMHC, respectively. This score was generated for each pMHC complex assessed.

### Training TITAN to examine TCR-epitope specificity

TITAN is a bimodal convolutional neural network [42] that encodes TCR and epitope sequences to predict the binding probability of TCR-epitope interactions. A limitation of TITAN is that it does not consider the HLA allele presenting an epitope. Therefore, predictions made using this model are interpreted and analysed in a ‘pan-peptide’ fashion.

For our work, we trained TITAN using the full TCR sequence: V, J gene sequences and CDR3. TCRs were encoded using BLOSUM62. Due to the superior performance in the authors’ original study, we encoded the peptides using SMILES, which is an atom-level description commonly used in chemoinformatics. Full retraining details are provided in the Supplementary Methods.

### Relative TCR promiscuity score (RTP)

TITAN was used to predict binding between epitope and TCR.


$$\begin{array}{l} RTP=log\left(\frac{\#TCRs\;\;bind\;\;Omicron\;epitope}{\#TCRs\;\;bind\;\;Wuhan\;epitope}\right) \end{array}$$

TITAN does not consider MHC. Therefore, RTP is computed for each peptide. RTP > 0 reflects increased breadth after Omicron mutation; simply, binding more TCRs. RTP should be considered as a ‘pan peptide’ score, while RI is peptide-MHC specific.

### Analysis of the MIRA dataset

The COVID-19 MIRA dataset was downloaded from https://clients.adaptivebiotech.com/pub/covid-2020 with sample metadata. These data contain TCR repertoire data mapped to SARS-CoV-2 epitopes from patient cohorts. As our analysis focused on how Omicron may affect T cell memory, healthy patients were excluded. Given our focus on CD8+ T cells, class II TCR-epitope samples were excluded. Unproductive TCRs were excluded. We define the ‘TCR-epitope’ repertoire, as each subject’s collection of unique TCR-epitope samples from their full TCR repertoire. While the MHC of each subject is often known, the MIRA dataset maps TCR-epitope and does not explicitly link TCR-epitope-MHC. In effect, by using these data, it is not always possible to infer the specific MHC linked to the specific TCR-epitope sample. Therefore, we analysed the MIRA data in a ‘pan-peptide’ manner. To do this, for each peptide, we took the average RI score across predicted bound MHC after Omicron, generating a single score for each immunogenic peptide with a mutation in Omicron. These data were then integrated with the MIRA TCR repertoires. Any peptides in the repertoires that are not mutated in the Omicron variant analysed were imputed with a ‘0’ RI score, reflecting no change.

### *In silico* mutagenesis

As TRAP is limited to 9- and 10-mers, we focused on single-point substitutions only. Immunogenicity scores for each wild-type peptide *i* (original Wuhan Hu-1 epitope) and its theoretical mutants (MT_*k*…_MT_*n*_, where *n* = 171 for 9-mers and 190 for 10-mers) against 64 HLA were generated as described previously.

To maximise sample size, for the *in silico* mutagenesis analysis only, we relaxed the threshold for excluding paired WT and MT pMHC from analysis (as we are only interested in when WT and/or MT are immunogenic) from <0.5 (as above, see ‘Comparing T cell immunogenicity’) to <0.35.

For panels A–D in Fig. 5, we analysed log ratios in a ‘pan-HLA’ manner. Thus, for each MT_*k*_, we then took the mean score across filtered MHC as their ‘immunogenicity score’. So, for each wild-type peptide *i,* we compared one score (its average across MHC), with *n* (*n* = 171 for 9-mers and 190 for 10-mers) MT scores (each representing their average across MHC).

To generate the ‘escape score’, we took the mean ± standard error of log ratios (MT/WT) across all substitutions and assessed MHC involving a particular epitope. The escape score was then multiplied by −1 to produce a score where >0 reflects escape opportunity. Therefore:


$$\begin{array}{l} Escape\;score=\log\left(\frac{MT}{WT}\right)\;\times -1 \end{array}$$

For Fig. 5F, we explored whether amino acid properties of wildtype peptides may contribute to their escape-prone status. To define visualised ‘high’ and ‘low’ groups, we selected the nine epitopes with the highest and lowest ‘escape scores’, respectively. For the ‘tolerant’ group, we selected nine epitopes with mean ± standard deviation around zero (i.e. limited change given the theoretical mutations).

### Neighbour networks

We adopted the approach and adapted the code from Ogishi *et al.* [44] and their Repitope package: https://github.com/masato-ogishi/Repitope. We generated a network-style representation of the single amino acid substitution trajectories, where pairs of peptide sequences with just one alteration are defined as neighbours and regarded as edges. Clustering was performed using a walk-trap algorithm from the R *igraph* package. We adapted the Repitope code for our own use case and to incorporate our own immunogenicity scores rather than those output from Repitope.

## Supplementary Material

ltad005_suppl_Supplementary_Material

ltad005_suppl_Supplementary_Data_1_Table_1

ltad005_suppl_Supplementary_Data_2_Table_2

ltad005_suppl_Supplementary_Data_3_Table_3

ltad005_suppl_Supplementary_Data_4_Table_4

ltad005_suppl_Supplementary_Data_5_Table_5

## Data Availability

Key datasets generated or analysed during this study are included in this published article, its supplementary information files or in a GitHub repository https://github.com/paulrbuckley/ImpactCoV2MutationsTcells. Code used to perform the analysis can be found on the aforementioned GitHub repository. The animal research: not applicable. Permission to reproduce: Only for scientific purposes with citations to this publication.

## Abbreviations

SARS-CoV-2: Severe acute respiratory syndrome coronavirus 2
VOC: Variants of concern
TRAP: T cell recognition potential of HLA-I presented peptides

## References

[1] Sigal A. Milder disease with Omicron: is it the virus or the pre-existing immunity? Nat Rev Immunol 2022;22:69–71. 10.1038/s41577-022-00678-4 35046570 PMC8767774

[2] Willett BJ , Grove J, MacLean OA et al. SARS-CoV-2 Omicron is an immune escape variant with an altered cell entry pathway. Nat Microbiol 2022;7:1161–79. 10.1038/s41564-022-01143-7 35798890 PMC9352574

[3] Cele S , Jackson L, Khoury DS et al. Omicron extensively but incompletely escapes Pfizer BNT162b2 neutralization. Nature 2022;602:654–6. 10.1038/s41586-021-04387-1 35016196 PMC8866126

[4] Cao Y , Wang J, Jian F et al. Omicron escapes the majority of existing SARS-CoV-2 neutralizing antibodies. Nature 2022;602:657–63. 10.1038/s41586-021-04385-3 35016194 PMC8866119

[5] Cameroni E , Bowen JE, Rosen LE et al. Broadly neutralizing antibodies overcome SARS-CoV-2 Omicron antigenic shift. Nature 2022;602:664–70. 10.1038/s41586-021-04386-2 35016195 PMC9531318

[6] Khan K , Karim F, Ganga Y et al. Omicron BA.4/BA.5 escape neutralizing immunity elicited by BA.1 infection. Nat Commun 2022;13:4686. 10.1038/s41467-022-32396-9 35948557 PMC9364294

[7] Gao Y , Cai C, Grifoni A et al. Ancestral SARS-CoV-2-specific T cells cross-recognize the Omicron variant. Nat Med 2022;28:472–6. 10.1038/s41591-022-01700-x 35042228 PMC8938268

[8] Grifoni A , Sette A. From Alpha to omicron: the response of T cells. Curr Res Immunol 2022;3:146–50. 10.1016/j.crimmu.2022.08.005 35966178 PMC9364680

[9] Mclean G , Kamil J, Lee B et al. The impact of evolving SARS-CoV-2 mutations and variants on COVID-19 vaccines. mBio 2022;13(2):e0297921. 35352979 10.1128/mbio.02979-21 PMC9040821

[10] Tarke A , Sidney J, Methot N et al. Impact of SARS-CoV-2 variants on the total CD4+ and CD8+ T cell reactivity in infected or vaccinated individuals. Cell Rep Med 2021;2. 10.1016/j.xcrm.2021.100355 PMC8249675 34230917

[11] Alison Tarke A , Sidney J, Methot N et al. Negligible impact of SARS-CoV-2 variants on CD4 + and CD8 + T cell reactivity in COVID-19 exposed donors and vaccines.

[12] Keeton R , Richardson SI, Moyo-Gwete T et al. Prior infection with SARS-CoV-2 boosts and broadens Ad26.COV2.S immunogenicity in a variant-dependent manner. Cell Host Microbe 2021;29:1611–19.e5. 10.1016/j.chom.2021.10.003 34688376 PMC8511649

[13] Melo-González F , Soto JA, González LA et al. Recognition of variants of concern by antibodies and T cells induced by a SARS-CoV-2 inactivated vaccine. Front Immunol 2021;12:4679. 10.3389/fimmu.2021.747830 PMC8630786 34858404

[14] Riou C , Keeton R, Moyo-Gwete T et al. Escape from recognition of SARS-CoV-2 variant spike epitopes but overall preservation of T cell immunity. Sci Transl Med 2022;14:6824. 10.1126/scitranslmed.abj6824 PMC9434381 34931886

[15] Geers D , Shamier MC, Bogers S et al. SARS-CoV-2 variants of concern partially escape humoral but not T-cell responses in COVID-19 convalescent donors and vaccines. Sci Immunol 2021;6:1750. 10.1126/sciimmunol.abj1750 PMC9268159 34035118

[16] Tarke A , Coelho CH, Zhang Z et al. SARS-CoV-2 vaccination induces immunological T cell memory able to cross-recognize variants from Alpha to Omicron. Cell 2022;185:847–59.e11. 10.1016/j.cell.2022.01.015 35139340 PMC8784649

[17] Naranbhai V , Nathan A, Kaseke C et al. T cell reactivity to the SARS-CoV-2 Omicron variant is preserved in most but not all individuals. Cell 2022. 10.1016/j.cell.2022.03.022 PMC8969090 35364034

[18] Reynolds CJ , Pade C, Gibbons JM et al. Immune boosting by B.1.1.529 (Omicron) depends on previous SARS-CoV-2 exposure. Science 2022:eabq1841. 35699621 10.1126/science.abq1841 PMC9210451

[19] Suryawanshi R , Ott M. SARS-CoV-2 hybrid immunity: silver bullet or silver lining? Nat Rev Immunol 2022:1–2. 10.1038/s41577-022-00771-8 PMC9362961 35945353

[20] Noval Rivas M , Porritt RA, Cheng MH et al. Multisystem inflammatory syndrome in children and long COVID: the SARS-CoV-2 viral superantigen hypothesis. Front Immunol 2022;13. 10.3389/fimmu.2022.941009 PMC9300823 35874696

[21] Klein J , Wood J, Jaycox J, et al. Distinguishing features of long COVID identified through immune profiling. medRxiv 2022. 10.1101/2022.08.09.22278592 PMC10620090 37748514

[22] Al-Aly Z , Bowe B, Xie Y. Long COVID after breakthrough SARS-CoV-2 infection. Nat Med 2022:1–7. 35614233 10.1038/s41591-022-01840-0 PMC9307472

[23] Brodin P , Arditi M. Severe acute hepatitis in children: investigate SARS-CoV-2 superantigens. Lancet Gastroenterol Hepatol 2022;7(7):594–5. 10.1016/S2468-1253(22)00166-2 PMC9106421 35576952

[24] Sacco K , Castagnoli R, Vakkilainen S et al. Immunopathological signatures in multisystem inflammatory syndrome in children and pediatric COVID-19. Nat Med 2022;28:1050–62. 10.1038/s41591-022-01724-3 35177862 PMC9119950

[25] Porritt RA , Paschold L, Rivas MN et al. HLA class I-associated expansion of TRBV11-2 T cells in multisystem inflammatory syndrome in children. J Clin Investig 2021;131. 10.1172/JCI146614 PMC8121516 33705359

[26] Hamdy A , Leonardi A. Superantigens and SARS-CoV-2. Pathogens 2022;11:390. 10.3390/pathogens11040390 35456065 PMC9026686

[27] Hongying Cheng M , Zhang S, Porritt RA, et al. Superantigenic character of an insert unique to SARS-CoV-2 spike supported by skewed TCR repertoire in patients with hyperinflammation. 10.1073/pnas.2010722117 PMC7568239 32989130

[28] Shen XR , Geng R, Li Q et al. ACE2-independent infection of T lymphocytes by SARS-CoV-2. Signal Transduct Target Ther 2022;7:1–11. 35277473 10.1038/s41392-022-00919-x PMC8914143

[29] Nersisyan S , Zhiyanov A, Zakharova M et al. Alterations in SARS-CoV-2 Omicron and Delta peptides presentation by HLA molecules. PeerJ 2022;10:e13354. 10.7717/peerj.13354 35502206 PMC9055995

[30] Foix A , López D, Díez-Fuertes F et al. Predicted impact of the viral mutational landscape on the cytotoxic response against SARS-CoV-2. PLoS Comput Biol 2022;18:e1009726. 10.1371/journal.pcbi.1009726 35143484 PMC8830725

[31] Pretti MAM , Galvani RG, Scherer NM et al. In silico analysis of mutant epitopes in new SARS-CoV-2 lineages suggest global enhanced CD8+ T cell reactivity and also signs of immune response escape. Infect Genet Evol 2022;99:105236. 10.1016/j.meegid.2022.105236 35149224 PMC8824006

[32] Dolton G , Rius C, Hasan MS et al. Emergence of immune escape at dominant SARS-CoV-2 killer T cell epitope. Cell 2022;185:2936–51.e19. 10.1016/j.cell.2022.07.002 35931021 PMC9279490

[33] Lee CH , Huh J, Buckley PR, et al. A robust deep learning platform to predict CD8+ T-cell epitopes. bioRxiv 2022. 10.1101/2022.12.29.522182

[34] Hamelin DJ , Fournelle D, Grenier JC et al. The mutational landscape of SARS-CoV-2 variants diversifies T cell targets in an HLA-supertype-dependent manner. Cell Syst 2022;13:143–57.e3. 10.1016/j.cels.2021.09.013 34637888 PMC8492600

[35] dos Santos Francisco R , Buhler S, Nunes JM et al. HLA supertype variation across populations: new insights into the role of natural selection in the evolution of HLA-A and HLA-B polymorphisms. Immunogenetics 2015;67:651–63. 10.1007/s00251-015-0875-9 26459025 PMC4636516

[36] Burns JC. MIS-C: myths have been debunked, but mysteries remain. Nat Rev Rheumatol 2023:1–2. 10.1038/s41584-022-00896-z PMC9817441 36609687

[37] Amormino C , Tedeschi V, Paldino G et al. SARS-CoV-2 spike does not possess intrinsic superantigen-like inflammatory activity. Cells 2022;11:2526. 10.3390/cells11162526 36010602 PMC9406418

[38] Lee CH , Antanaviciute A, Buckley PR. To what extent does MHC binding translate to immunogenicity in humans? ImmunoInformatics 2021;3–4:100006.

[39] Buckley PR , Lee CH, Ma R et al. Evaluating performance of existing computational models in predicting CD8+ T cell pathogenic epitopes and cancer neoantigens. Brief Bioinform 2022;23:1–18. 10.1093/bib/bbac141 PMC9116217 35471658

[40] Motozono C , Toyoda M, Tan TS et al. The SARS-CoV-2 Omicron BA.1 spike G446S mutation potentiates antiviral T-cell recognition. Nat Commun 2022;13:1–11. 36130929 10.1038/s41467-022-33068-4 PMC9492656

[41] Li Y , Wang X, Jin J et al. T-cell responses to SARS-CoV-2 Omicron spike epitopes with mutations after the third booster dose of an inactivated vaccine. J Med Virol 2022;94:3998–4004. 10.1002/jmv.27814 35474581 PMC9088599

[42] Nolan S , Vignali M, Klinger M et al. A large-scale database of T-cell receptor beta (TCRβ) sequences and binding associations from natural and synthetic exposure to SARS-CoV-2. Res Sq 2020:1–28. 10.3389/fimmu.2025.1488851 PMC11873104 40034696

[43] Ogishi M , Yotsuyanagi H. Quantitative prediction of the landscape of T cell epitope immunogenicity in sequence space. Front Immunol 2019;10:827. 10.3389/fimmu.2019.00827 31057550 PMC6477061

[44] DeGrace MM , Ghedin E, Frieman MB et al. Defining the risk of SARS-CoV-2 variants on immune protection. Nature 2022;605:640–52. 10.1038/s41586-022-04690-5 35361968 PMC9345323

[45] Babady NE , Burckhardt RM, Krammer F et al. Building a resilient scientific network for COVID-19 and beyond. Am Soc Microbiol 2022;13:e0222322. 10.1128/mbio.02223-22 PMC9600431 36125317

[46] Maher MC , Bartha I, Weaver S et al. Predicting the mutational drivers of future SARS-CoV-2 variants of concern. Sci Transl Med 2022;14:3445. 10.1126/scitranslmed.abk3445 PMC8939770 35014856

[47] Flemming A. Omicron, the great escape artist. Nat Rev Immunol 2022;22:75. 10.1038/s41577-022-00676-6 35017722 PMC8749340

[48] Choi SJ , Kim D-U, Noh JY et al. T cell epitopes in SARS-CoV-2 proteins are substantially conserved in the Omicron variant. Cell Mol Immunol 2022:1–2. 10.1038/s41423-022-00838-5 PMC8764507 35043006

[49] Mazzoni A , Vanni A, Spinicci M et al. SARS-CoV-2 spike-specific CD4+ T cell response is conserved against variants of concern, including omicron. Front Immunol 2022;13:121. 10.3389/fimmu.2022.801431 PMC8826050 35154116

[50] Tarke A , Coelho CH, Zhang Z et al. SARS-CoV-2 vaccination induces immunological T cell memory able to cross-recognize variants from Alpha to Omicron. Cell 2022;185(5):847–59.e11. 35139340 10.1016/j.cell.2022.01.015 PMC8784649

[51] Martinez-Sobrido L , Almazan Toral F, Faraz Ahmed S et al. SARS-CoV-2 T cell responses elicited by COVID-19 vaccines or infection are expected to remain robust against omicron. Viruses 2022;14:79. 35062283 10.3390/v14010079 PMC8781795

[52] Keeton R , Tincho MB, Ngomti A et al. T cell responses to SARS-CoV-2 spike cross-recognize Omicron. Nature 2022;603:488–92. 10.1038/s41586-022-04460-3 35102311 PMC8930768

[53] Grifoni A , Sidney J, Vita R et al. SARS-CoV-2 human T cell epitopes: adaptive immune response against COVID-19. Cell Host Microbe 2021. 10.1016/j.chom.2021.05.010 PMC8139264 34237248

[54] Grifoni A , Weiskopf D, Ramirez SI et al. Targets of T cell responses to SARS-CoV-2 coronavirus in humans with COVID-19 disease and unexposed individuals. Cell 2020;181:1489–501.e15. 10.1016/j.cell.2020.05.015 32473127 PMC7237901

[55] Tarke A , Sidney J, Methot N et al. Impact of SARS-CoV-2 variants on the total CD4^+^; and CD8^+^; T cell reactivity in infected or vaccinated individuals. 2021. 10.1016/j.xcrm.2021.100355 PMC8249675 34230917

[56] Kedzierska K , Thomas PG. Count on us: T-cells in SARS-CoV-2 infection and vaccination. Cell Rep Med 2022;3(3);100562. 35474748 10.1016/j.xcrm.2022.100562 PMC8872824

[57] Stanevich O , Alekseeva E, Sergeeva M et al. SARS-CoV-2 escape from cytotoxic T cells during long-term COVID-19. Res Sq 2022. 10.1038/s41467-022-34033-x PMC9831376 36627290

[58] Yu F , Tai W, Cheng G. T-cell immunity: a barrier to Omicron immune evasion. Signal Transduct Target Ther 2022;7:1–3. 36031636 10.1038/s41392-022-01142-4 PMC9420176

[59] Alwan NA. Lessons from Long COVID: working with patients to design better research. Nat Rev Immunol 2022;22:201–2. 10.1038/s41577-022-00692-6 35169259 PMC8853146

[60] Galán M , Vigón L, Fuertes D et al. Persistent overactive cytotoxic immune response in a Spanish cohort of individuals with Long-COVID: identification of diagnostic biomarkers. Front Immunol 2022;13:848886. 35401523 10.3389/fimmu.2022.848886 PMC8990790

[61] Szabo PA , Dogra P, Gray JI et al. Longitudinal profiling of respiratory and systemic immune responses reveals myeloid cell-driven lung inflammation in severe COVID-19. Immunity 2021;54:797–814.e6. 10.1016/j.immuni.2021.03.005 33765436 PMC7951561

[62] He J , Cai S, Feng H et al. Single-cell analysis reveals bronchoalveolar epithelial dysfunction in COVID-19 patients. Protein Cell 2020;11:680–7. 10.1007/s13238-020-00752-4 32671793 PMC7363016

[63] Jariani A , Warth C, Deforche K et al. SANTA-SIM: simulating viral sequence evolution dynamics under selection and recombination. Virus Evol 2019;5. 10.1093/ve/vez003 PMC6407609 30863552

[64] Weber A , Born J, Rodriguez Martínez M. TITAN: T-cell receptor specificity prediction with bimodal attention networks. Bioinformatics 2021;37:i237–44. 10.1093/bioinformatics/btab294 34252922 PMC8275323

